# Review and Current Perspectives on DNA Topoisomerase I and II Enzymes of Fungi as Study Models for the Development of New Antifungal Drugs

**DOI:** 10.3390/jof10090629

**Published:** 2024-09-03

**Authors:** Dulce Andrade-Pavón, Omar Gómez-García, Lourdes Villa-Tanaca

**Affiliations:** 1 Laboratorio de Biología Molecular de Bacterias y Levaduras, Departamento de Microbiología, Escuela Nacional de Ciencias Biológicas, Instituto Politécnico Nacional, Prol. de Carpio y Plan de Ayala. Col. Sto. Tomás, Ciudad de México 11340, Mexico; mvillat@ipn.mx; 2 Departamento de Fisiología, Escuela Nacional de Ciencias Biológicas, Instituto Politécnico Nacional, Av. Wilfrido Massieu 399, Nueva Industrial Vallejo, Gustavo A. Madero, Ciudad de México 07738, Mexico; 3 Departamento de Química Orgánica, Escuela Nacional de Ciencias Biológicas, Instituto Politécnico Nacional, Prol. de Carpio y Plan de Ayala. Col. Sto. Tomás, Ciudad de México 11340, Mexico

**Keywords:** fungus, fungal infections, DNA topoisomerases, Topo I, Topo II, Topo I and II inhibitors, antifungals, molecular target

## Abstract

Fungal infections represent a growing public health problem, mainly stemming from two phenomena. Firstly, certain diseases (e.g., AIDS and COVID-19) have emerged that weaken the immune system, leaving patients susceptible to opportunistic pathogens. Secondly, an increasing number of pathogenic fungi are developing multi-drug resistance. Consequently, there is a need for new antifungal drugs with novel therapeutic targets, such as type I and II DNA topoisomerase enzymes of fungal organisms. This contribution summarizes the available information in the literature on the biology, topology, structural characteristics, and genes of topoisomerase (Topo) I and II enzymes in humans, two other mammals, and 29 fungi (including Basidiomycetes and Ascomycetes). The evidence of these enzymes as alternative targets for antifungal therapy is presented, as is a broad spectrum of Topo I and II inhibitors. Research has revealed the genes responsible for encoding the Topo I and II enzymes of fungal organisms and the amino acid residues and nucleotide residues at the active sites of the enzymes that are involved in the binding mode of topoisomerase inhibitors. Such residues are highly conserved. According to molecular docking studies, antifungal Topo I and II inhibitors have good affinity for the active site of the respective enzymes. The evidence presented in the current review supports the proposal of the suitability of Topo I and II enzymes as molecular targets for new antifungal drugs, which may be used in the future in combined therapies for the treatment of infections caused by fungal organisms.

## 1. Introduction

Fungal infections represent an emerging disease that has become a public health problem worldwide, causing high rates of morbidity and mortality in hospitalized patients with an immune system weakened by a disease and/or treatment [1,2,3,4,5,6]. Whereas fungi of the genus *Candida* [7,8,9], especially *Candida albicans* [10,11,12], used to be the principal agent of hospital infections, in recent years, new multi-drug-resistant pathogens have emerged, such as *C. auris, C. haemulonii* [13,14,15,16,17], and non-*Candida* fungi (e.g., *Aspergillus* spp., *Fusarium* spp., and species of the order Mucorales) [18,19,20]. These pathogens have claimed the lives of patients in intensive care units, including those infected with the SARS-CoV-2 virus [21,22,23,24,25,26,27,28,29].

Various mechanisms of drug resistance have been identified for pathogenic fungi, including the mutation and/or overexpression of the enzyme targeted by the antifungal agent, an alteration in the enzymes related to the synthesis of ergosterol, the creation of efflux pumps of the ATP-binding cassette (ABC) or major facilitator (MF) superfamilies, and the formation of biofilms [30,31,32,33]. Indeed, several strains of *Candida* have developed resistance to polyenes, azoles, and echinocandins, representing all three classes of antifungal drugs prescribed to eliminate them [8,34,35,36,37,38]. Likewise, cases exist of clinical isolates of *Aspergillus*, *Fusarium*, and the causal agents of mucormycosis with the same pattern of multi-drug resistance [39,40,41,42,43,44,45].

Due to the drug resistance of a growing number of pathogenic fungi, there is an urgent need to find more effective antifungal treatments with novel targets. The DNA topoisomerase (Topo) I and II enzymes of fungi have been proposed as plausible alternative targets for drug therapy [46,47,48]. The current contribution is a bibliographic review of the information available on the biology, topology, structural characteristics, DNA sequences, and genes of Topo I and II enzymes in humans, two other mammals, and 29 fungal organisms (including Basidiomycetes and Ascomycetes). The evidence of these enzymes as alternative targets for antifungal therapy is presented, as is a broad spectrum of Topo I and II inhibitors. Research has revealed the genes responsible for encoding the Topo I and II enzymes of fungal organisms, and the amino acid residues and nucleotide residues at the active site of the enzymes that are involved in the binding of known topoisomerase inhibitors. According to molecular docking studies, antifungal Topo I and II inhibitors have good affinity for the active site of the respective enzymes. It should be possible to design and synthesize analogues of such inhibitors with structural modifications capable of improving their efficacy on a wide variety of fungal organisms and reducing their toxicity. The current contribution aims to emphasize the potential of Topo I and II enzymes as targets for the design of new antifungal drugs and to explore the differences between the two enzymes.

## 2. DNA Topoisomerases: Definition and Classification

DNA topoisomerase enzymes are able to solve topological problems stemming from the replication, transcription, recombination, and remodeling of DNA chromatin in cells. Topo I and II are two large groups of enzymes that perform this function by allowing for the introduction of breaks in one or both DNA strands [49,50,51,52,53]. They are highly conserved in the three domains of cellular life (Archaea, Bacteria, and Eukarya) and in viruses [54]. They are called Topo I and II because Topo I cuts one strand of DNA and Topo II cuts both strands of the DNA chain. DNA topoisomerases are divided into two classes: type I enzymes (topoisomerases I, III and V) and type II enzymes (topoisomerases II, IV and VI) [52].

Topo I, a monomeric enzyme, relaxes positively or negatively supercoiled DNA without requiring energy from ATP. It breaks only one strand of DNA and only relaxes supercoiled DNA. Topo I enzymes are subdivided into two groups, depending on whether they form the covalent enzyme–substrate intermediate with the 5′ end (IA) or the 3′ end (IB) of the cleaved segment. The intermediate is generated at the active site of the enzyme by a nucleophilic attack of the hydroxyl group of a tyrosine on a phosphate group of the nucleotide chain, creating a transient phosphodiester bond capable of breaking the DNA chain.

Topo II enzymes exist in both eukaryotic and prokaryotic organisms. Eukaryotes express Topo IIA, the essential and main type of the enzyme. This enzyme participates in the relaxation of DNA in a negative and positive way in the presence of ATP and Mg^2+^. The Topo II enzyme in yeasts is a homodimer and plays a role in the relief of torsional strain during DNA replication, with a preference for the relaxation of positive supercoils ahead of the fork. In addition, yeast Topo II supports the transcription of long genes (>3 kb), and its absence stalls fork progression, which cannot be rescued by Topo I [55,56,57].

The two isoforms of Topo II in vertebrates are Topo IIα and IIβ. They have almost 77% sequence homology, differing in the C-terminal region. In particular, the C-terminus of Topo IIα shifts the activity of the enzyme toward the preferential relaxation of positive supercoils, whereas the equivalent region of Topo IIβ does not appear to express any supercoil preference. These differences may be linked to particular cellular functions. Topo IIα is critical for cellular viability and plays essential roles during DNA replication, mitosis, cell-cycle regulated expression, chromosome condensation, segregation, and replication. On the other hand, Topo IIβ is associated with the repair, transcription, and development of DNA [58,59].

In bacteria, Topo II is subdivided into Topo IV and DNA gyrase. The former utilizes the hydrolysis of ATP to relax supercoiled DNA, decatenate replication products, and knot and unknot DNA. DNA gyrase introduces supercoils in the double-stranded DNA chain in the presence of ATP [60,61,62]. Topo VI was first detected in the archaeal hyperthermophile *Sulfolobus shibatae* and has since been found throughout the archaea, in a few bacterial species and, intriguingly, in eukaryotes such as plants and algae [62,63].

Topo III, a well-conserved enzyme in prokaryotes, eukaryotes, and archaea, is a type IA topoisomerase that relaxes and decatenates DNA but also has the ability to cleave and decatenate RNA molecules. Topo V has been described as a type IB topoisomerase as it shows similarities to eukaryotic topo I and can relax negatively and positively supercoiled DNA through a controlled rotation/swivel mechanism by nicking one strand on the DNA and allowing the other strand to rotate around it. To date, it has been found in only one genus of Archaea (*Methanopyrus*) [52].

## 3. Characteristics of the Genes That Code for Topo I and II of Fungal Organisms

The sequences of the amino acids in Topo I and Topo II were downloaded from the NCBI database, and the genes of these enzymes were identified for each of the 29 fungi and three mammals (*Homo sapiens*, *Mus musculus*, and *Rattus norvegicus*) herein examined. The DNA sequences were determined, and the ones corresponding to *H. sapiens* were compared to those of the other 31 organisms (Table 1), with one representative organism chosen for Ascomycetes (*Candida auris*) and one for Basidiomycetes (*Cryptococcus neoformans*). The comparison confirmed the existence of Topo Iβ and Topo II⍺ in all thirty-two organisms. In *Saccharomyces cerevisiae* and *Schizosaccharomyces pombe*, the genes were identified that code for Topo I, which is dispensable for growth, and for Topo II, which is absolutely necessary to “decatenate” linked chromosomes and prepare chromosomes for segregation at mitosis. Topo II in *S. cerevisiae* is reportedly regulated by the cell cycle [51,52]. Unlike yeast cells, most mammalian cells appear to contain two type II topoisomerases isoforms, termed IIα and IIβ [51].

The amino acid sequences of the Topo Iβ and Topo II⍺ enzymes of *H. sapiens* were compared to the corresponding sequences of the other thirty-one organisms currently under study, finding from 28.6 to 100% identity and similarity (Tables S1 and S2, Supplementary Materials). These values were obtained from the alignment of two sequences of interest on the EMBOSS Water-Smith server (https://www.ebi.ac.uk/jdispatcher/psa/emboss_water) (accessed on 1 July 2024).

**Figure 1:**
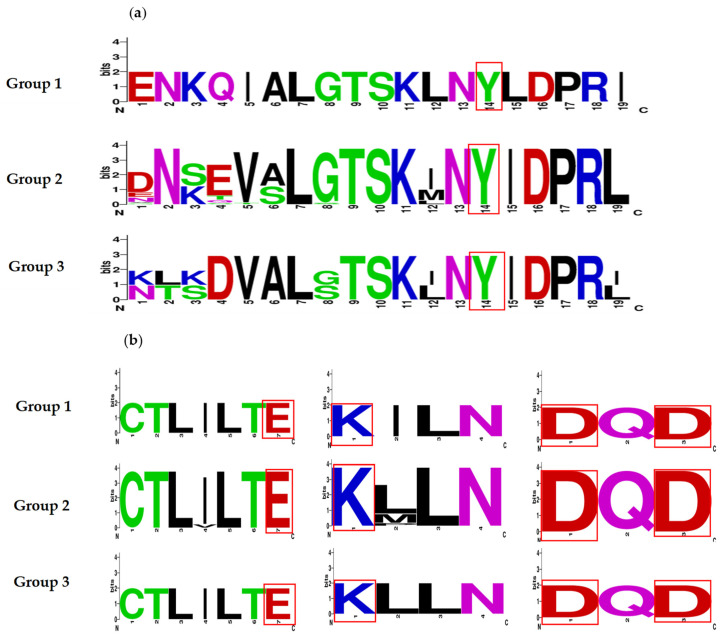
Sequence logos of the conserved motifs found in topoisomerase I (**a**) and II (**b**) enzymes pertaining to the families of the organisms under study. Group 1 consists of mammals (*Homo sapiens*, *Mus musculus*, and *Rattus norvegicus*), group 2 of Ascomycetes, and group 3 of Basidiomycetes. The catalytic amino acid residues involved in the mechanism of action are highlighted in red boxes.

The active site was identified for Topo I and Topo II by means of multiple sequence alignment (Figure S1, Supplementary Material) on the Clustal Omega program (https://www.ebi.ac.uk/jdispatcher/msa/clustalo) (accessed on 1 July 2024), as were the catalytic amino acid residues involved in the mechanism of action. Such residues proved to be highly conserved in the three groups of organisms herein studied (mammals, Ascomycota, and Basidiomycota) (Figure 1a,b), as described in previous reports [47,64,65,66,67].

## 4. Biology of DNA Topoisomerase I and II Enzymes of Fungal Organisms

Strains of *C. albicans* with a deletion in the Topo I gene are deficient in their ability to form germ tubes and grow hyphae after serum induction in vitro. The depletion of the protein under conditions of promoter repression revealed that the Topo I gene is not essential for the in vitro growth of *C. albicans*. Nevertheless, the morphological changes stemming from the cultivation of *C. albicans* in a minimal medium showed an important role of Topo I in cellular processes [68]. Additionally, when *C. albicans* strains with a Topo I deletion were administered to mice, some attenuation of virulence was detected. With the deletion of both copies of the enzyme, a more pronounced attenuation was observed [68].

Similarly, the Topo I and II enzymes of *S. cerevisiae* have proven to be non-essential for the viability of yeast. One study demonstrated the viability of mutants of Topo I and II in which 1.7 kb of each of the sequences was deleted [69]. During temperature-shift experiments using cells from a single mutant (affecting Topo II), mitotic blocks were able to prevent cell death at a nonpermissive temperature. However, during the same treatment of cells from a double mutant (affecting both Topo I and II), mitotic blocks were ineffective in preventing cell death [69]. These findings suggest that in yeast cells, Topo I plays an auxiliary role in the function of Topo II. Moreover, another study demonstrated the viability of seven top1 (mak1) mutants, including those with gene disruptions. Thus, DNA topoisomerase I is not essential for yeast viability [70]. According to the first study carried out with *S. pombe*, two type I topoisomerase mutants were found to be viable. The second study conducted on the same yeast strain established the viability of a null strain made by Topo I gene disruption, although the generation time turned out to be 20% longer for the null versus wild-type strain [71,72].

In *S. cerevisiae* strains with a null mutation in the Topo I or Topo II gene, there is a 50- to 200-fold higher frequency of mitotic recombination in the ribosomal DNA (rDNA) pool compared to positive controls. Consequently, both Topo I and Topo II are required for the suppression of recombination in this region of the genome [73]. In Topo I from *S. cerevisiae*, the substitution of conserved residues in active site tyrosine (Tyr-727) produces alterations in the sensitivity of the yeast to camptothecin, while the substitution of alanine (A) by threonine (T) at position 722 in Topo I (T722A) enhances the stability of the DNA covalent intermediate. The cytotoxicity of camptothecin for Topo I T722A was attributed to its capacity to decrease DNA religation. The greater covalent complex formation of N726H in Topo I was ascribed to a relative increase in the rate of DNA cleavage [74]. Interestingly, the interaction of proline (P) A653P (used to substitute alanine) with the T718A mutation at the active site of Topo I suppresses the lethal phenotype caused by the T718A substitution but does not restore enzyme sensitivity to camptothecin. The specific activity of the double mutant is diminished in vivo and in vitro, consistent with a decline in DNA binding [75].

Furthermore, Topo I disruption delays the induction of a double-strand break (DSB) and shortens the window of this occurrence. In contrast, temperature-sensitive Topo II mutants are characterized by an elevated level of DSB signals on synapsed chromosomes and a marked delay in meiotic chromosome remodeling. These findings reveal an independent function of Topo I and II in the modulation of the meiotic chromosome structure and recombination [76].

When a top1 gene disruption mutant was constructed in *U. maydis*, the robust topoisomerase I activity found in wild-type *U. maydis* was lost, and a subtle coloration phenotype became evident during cell senescence [77], as previously described for *S. cerevisiae* [78].

Finally, when a second functional copy of the Topo I gene was introduced into the genome of *C. neoformans*, the yeast could be readily disrupted by homologous recombination. On the other hand, after the fusion of the Topo I gene to the GAL7 gene promoter, the GAL7::TOP1 fusion gene was modestly regulated by the carbon source in a serotype A strain of *C. neoformans*. The resulting overexpression of Topo I conferred sensitivity to heat shock, gamma-rays, and camptothecin. In an animal model of cryptococcal meningitis, Topo I regulation was not crucial for the establishment of an infection but might have influenced the initial stress response of the host. As can be appreciated, Topo I is essential in the human pathogen *C. neoformans* [79].

## 5. Structural Characteristics of DNA Topoisomerase I Enzymes of Fungal Organisms

**Figure 2:**
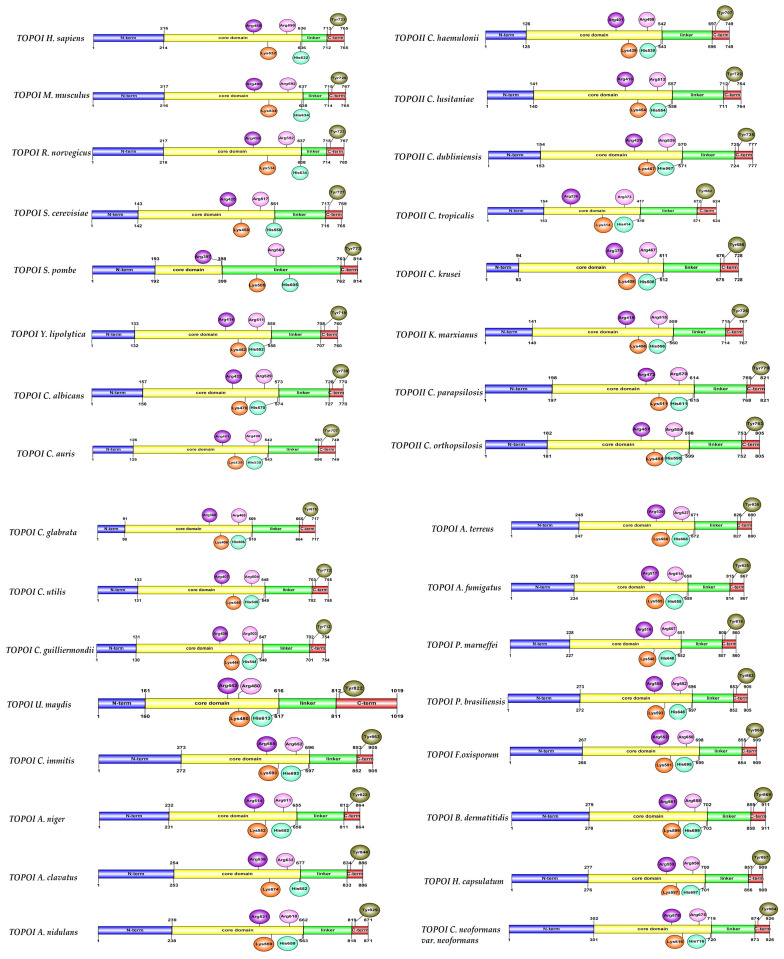
Schematic representation of Topo I enzymes from 32 organisms. Structural studies on the human enzyme divide this protein into four structural domains: the N-terminal hydrophilic domain (blue), the hydrophobic central “core” domain (yellow), the linker domain (green), and the C-terminal domain (red). The core domain interacts with DNA, and the linker domain connects the core with the catalytic site. The amino acids that are part of the catalytic site are portrayed in circles: purple (Arg), orange (Lys), pink (Arg), cyan (His), and green (Tyr).

**Figure 3:**
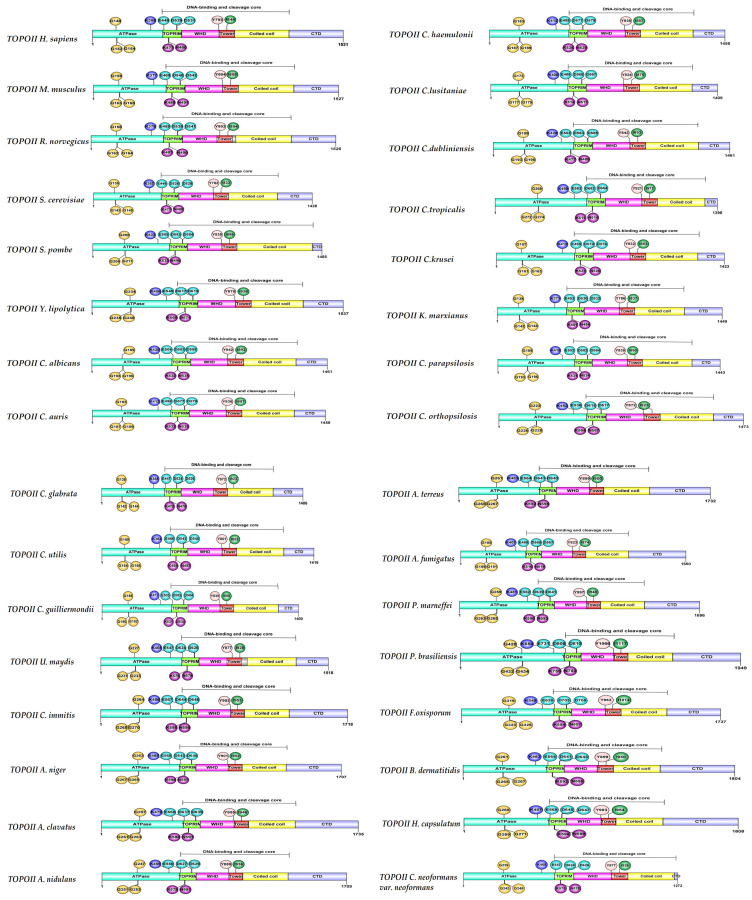
Schematic representation of Topo II enzymes from 32 organisms. The following domains are shown: ATPase (cyan), toprim (topoisomerase-primase, green), WHD (winged-helix domain, fuchsia), tower (red), coiled coil (yellow), and CTD (C-terminal domain, purple). The amino acids that are part of the catalytic site (Gly, Lys, Glu, Aps, Tyr, and Ile) are portrayed in circles of distinct colors.

A similarity was found when comparing the structural characteristics of the Topo I enzymes of *H. sapiens* and the 29 fungal organisms presently analyzed. There was also similarity between the corresponding Topo II enzymes (Figure 2 and Figure 3). The human Topo I gene encodes a protein with 765 amino acids and a theoretical molecular mass of 91 kDa. Crystallographic studies [51,52] have revealed four structural domains in the human protein (N-terminus, core, linker, and C-terminus). The N-terminal domain is hydrophilic in nature, has nuclear localization signals, and is dispensable for the in vitro activity of Topo I (as is the linker domain). It is not highly conserved among eukaryotic organisms, as the length of its sequences shows great variability. The core domain is linked to the N-terminus and the linker.

In the linker of the human protein, there are predominantly hydrophobic amino acids, with the remaining amino acids being hydrophilic. This region of the enzyme may exhibit amphipathic properties. It binds the core with the C-terminal domain, which contains the catalytic center involved in transiently cutting one of the DNA strands [51,52]. Four of the five amino acid residues at the catalytic site of the enzyme are located in the core domain (Arg488, Lys532, Arg590, and His632), while residue Tyr723 is in the C-terminal domain. The latter residue forms a covalent bond with the phosphodiester backbone of the cleaved single strand of DNA. According to one report, Tyr727 plays a similar role in *S. cerevisiae* [51,52,80,81,82]. The aforementioned characteristics of Topo I of *H. sapiens* are observed in the same enzyme of all twenty-nine fungal organisms examined presently (Figure 2). Since the architecture of these enzymes is highly conserved, the mechanisms of action of inhibitors of human Topo I are probably similar to those of inhibitors of fungal Topo I [51].

## 6. Structural Characteristics of DNA Topoisomerase II Enzymes of Fungal Organisms

Crystallographic studies have shown Topo II enzymes in yeasts to be homodimers. Each monomer has three functional domains, which closely correspond to those established in Topo II of *S. cerevisiae* and *S. pombe* [66].

The N-terminal domain has an ATPase function and is the most conserved among species. Its residues participate in dimer contacts. Toprim (topoisomerase-primase) is a structurally conserved catalytic domain of ~100 amino acids involved in DNA strand breakage and rejoining. It has two conserved motifs, one centered at a conserved glutamate and the other at two conserved aspartates (D × D). These residues are highly conserved in the Topo II enzymes of fungal organisms (Figure 3). Both motifs are preceded by conserved hydrophobic regions thought to form β-strands. A role is probably played by the glutamate residue in catalysis and by the D × D motif in the coordination of Mg^2+^ (required for the activity of all Toprim-containing enzymes) [83,84,85,86].

Topo II enzymes have a DNA binding/cleavage domain, a coiled-coil domain, and the C-terminal domain (CTD). The catalytic domain has two subdomains, termed the winged-helix domain (WHD) and the tower domain. The WHD is a five-helix bundle-bearing catalytic tyrosine, which is strictly conserved. The tower domain has a compact “spire” with two antiparallel β-strands packed against a four-helix bundle and a loose “base” consisting of mixed structural elements [52,65,83,85,86,87].

## 7. Phylogenetic Analysis of Type I and II DNA Topoisomerases of Fungal Organisms

According to phylogenetic studies, the main families of DNA topoisomerases are not homologous, indicating their independent origin. However, some of them share homologous subunits, which were probably recruited independently to bring about needed topoisomerase activities. Topo I and II enzymes have been found in all currently sequenced eukaryotic genomes (including fungal organisms), suggesting the presence of both in the last common eukaryotic ancestor [54,88,89].

A phylogenetic analysis of the Topo II enzyme sequences of *Candida* species demonstrated the close relationship of some species (*C. albicans*, *C. dubliniensis*, *C. parapsilosis*, and *C. tropicalis*) in relation to the ADPT cluster and of other species (*S. cerevisiae*, *C. kefyr*, and *C. glabrata*) in regard to the SCGK cluster. This same pattern has been described in other reports on Topo II of *Candida* spp. [90]. A phylogenetic study carried out on dermatophytes also evidenced the Topo II enzyme sequence [91].

**Figure 4:**
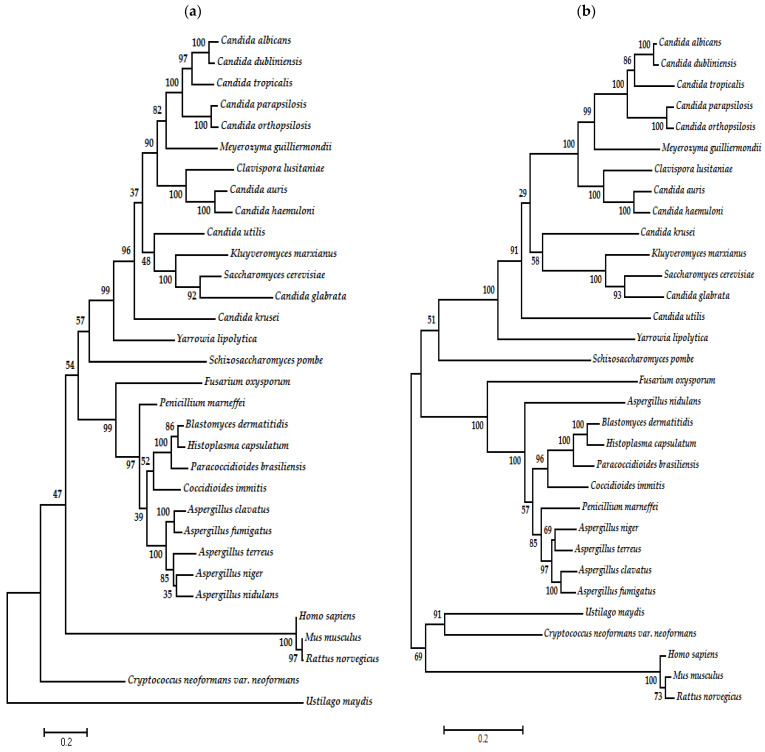
The maximum likelihood phylogenetic tree of 32 representative DNA topoisomerases of type I (**a**) and II (**b**) from mammals and fungi, constructed with the MEGA 6 program and the WAG + G model (bootstrap values are on the branches, out of 100 runs).

With the sequences of Topo I and II enzymes, two phylogenetic trees of fungal organisms of medical importance were constructed (Figure 4a,b), observing that some species of *Candida* are more related. Certain Ascomycetes (*Candida*, *Saccharomyces*, *Aspergillus*, *Blastomyces*, *Histoplasma*, *Penicillium*, *Fusarium*, *Coccidioides*, and *Paracoccidioides*) and Basidiomycetes (*Cryptococcus* and *Ustilago*) have a close relationship with respect to both Topo I and Topo II. The Topo I and II enzymes of mammals have greater similarity to the same enzymes of Basidiomycetes than those of Ascomycetes.

## 8. Topo I and II Enzymes of Fungi as Targets for Antifungal Therapy

Topo I and Topo II enzymes were proposed as targets for the discovery of new antifungal drugs for the first time in a report on the opportunistic pathogenic yeast *C. albicans* [81,82,83]. This species was isolated and purified through column procedures, followed by the determination of its enzymatic activity and its inhibition by known inhibitors of Topo I (camptothecin) and Topo II (etoposide and its derivatives). An inhibitory effect on the yeast was observed [47,92].

Heterologous expression studies have been conducted for Topo I and II enzymes. The Topo I gene of *C. albicans* has been isolated, cloned, and expressed in *S. cerevisiae* (a yeast of biotechnological interest), and the recombinant protein obtained was considered a tool for the development of therapeutic agents with antifungal activity [46,68,93]. Similar cloning and characterization studies have been carried out for the gene that codes for the Topo I enzyme of *A. nidulans* and *U. maydis* as well as the Topo II enzyme of *A. nidulans*. The corresponding recombinant proteins were also employed as tools for testing antifungal agents against the respective enzymes [77,94,95].

Consequently, yeast cells have been suggested as an ideal eukaryotic model for evaluating the effects of inhibitors of type I and II DNA topoisomerases [96,97,98,99,100]. Mutant yeast cells lacking Topo I are reported to be highly resistant to camptothecin, while those lacking Topo II are highly resistant to etoposide and amsacrine. Camptothecin and agents targeting Topo II of yeast cells reportedly act as topoisomerase poisons.

*S. cerevisiae* has been utilized as a prototype to establish Topo I and II enzymes as molecular targets and provide information on the activity of topoisomerase inhibitors [100,101,102].

**Figure 5:**
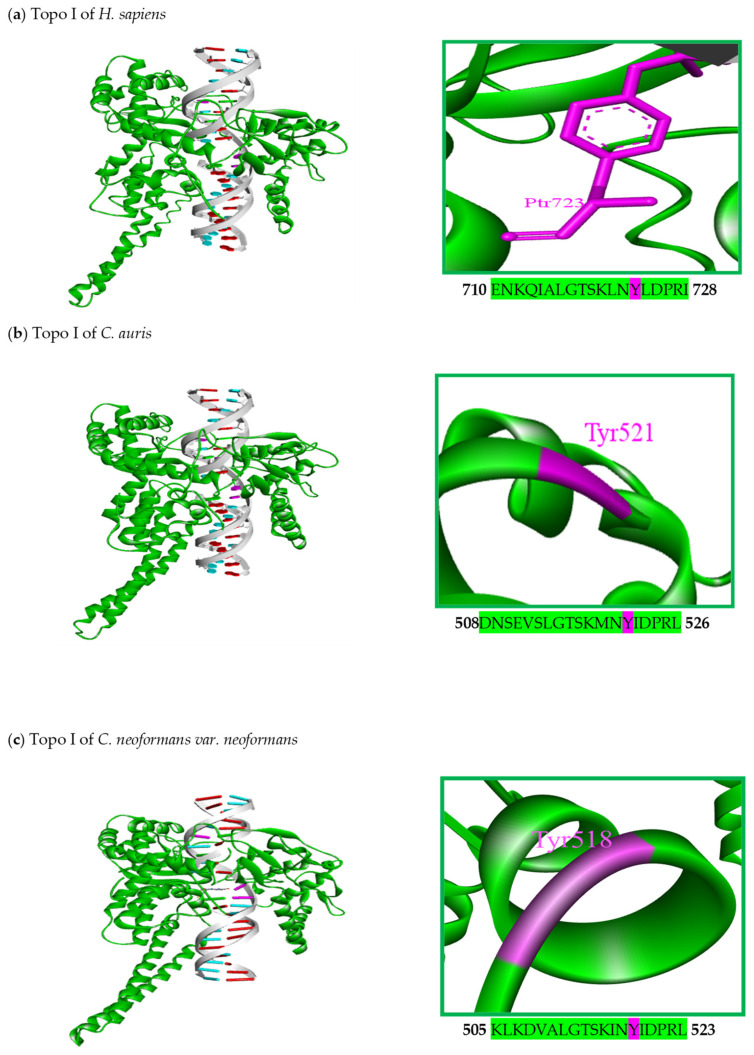

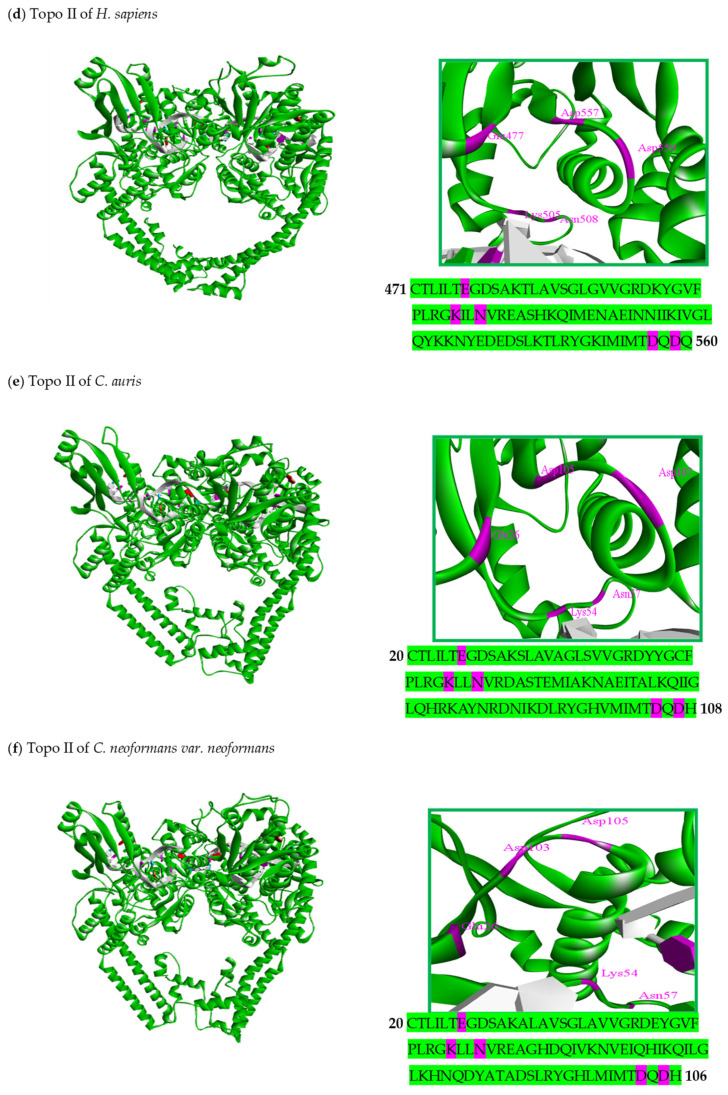
Flat ribbon illustration of the 3D models of DNA of topoisomerase I (**a**–**c**) and DNA topoisomerase II (**d**–**f**) in complex with the DNA of the representative organism. The active site amino acids of each of the proteins are highlighted in fuchsia. For the construction of the Topo I and Topo II models of *C. auris* and *C. neoformans*, the crystallized structures of Topo I (PDB: 1sc7) and Topo II (PDB: 3qx3) from *H. sapiens* were used as templates.

Based on studies that compare the amino acid sequences from Topo I and II, the 3D structure of the proteins can be predicted. The 3D structures were obtained for the Topo I and II enzymes of the 29 fungal organisms and three mammals herein included. The models were built with the homology modeling technique on the MODELLER program (1 July 2024) [101]. The crystallized proteins of Topo I (PDB: 1sc7) and Topo II (PDB: 3qx3) from *H. sapiens* in complex with a DNA chain of 22 base pairs were downloaded from the protein data bank (PDB, http://www.rcsb.org/) (accessed on 1 July 2024) and served as the template. The modeled protein was analyzed by the PROCHEK server to verify its quality (https://www.ebi.ac.uk/thornton-srv/software/PROCHECK/) (accessed on 1 July 2024). For the purpose of clarity, Figure 5a–f portray the 3D models of Topo I and II for only one representative organism of Ascomycetes (*Candida auris*), Basidiomycetes (*C. neoformans*), and mammals (*H. sapiens*). The amino acid sequence for the active site of each enzyme is shown, as are the amino acid residues involved in catalysis. Both the sequence and the residues are conserved. The overlapping of the models (Figure S1a,b, Supplementary Material) reveals the high structural identity between them. The quality of the model was evaluated by generating Ramachandran diagrams (Figure S2, Supplementary Material), as in other studies [47,102]. The quality was confirmed by the finding of up to 90% of the amino acid residues in favorable areas. Likewise, the calculation of the value of the root mean square deviation (RMSD) of the models (Table S3, Supplementary Material) resulted in values less than 1.2 Angstrom, which also indicates the quality of the models. Since the respective proteins have not yet been crystallized, the generation of these 3D models was very important to facilitate molecular docking studies, which were used to predict the binding mode and interaction energies between the DNA topoisomerases of fungal organisms and Topo I and II inhibitors.

On the right side of each model, there is an illustration of the active site (green) and the amino acid residues involved in coupling (fuchsia). A great similarity can be appreciated between the structural domains of the Topo I and II enzymes of the three families, evidencing the likelihood of a similar mechanism of action of their corresponding inhibitors. Hence, Topo I and II seem to be suitable targets for the design and development of new antifungal agents [79].

## 9. Selected Inhibitors of Topo I and II and Their Mechanism of Action

The two main classes of Topo I inhibitors are poisons and suppressors [103]. Poisons cause DNA strand breaks through the stabilization of topoisomerase II covalently bound to DNA in the intermediate form (the cleavable complex). Suppressors impede mitotic recombination processes by relaxing negative superhelicity [103].

Sensitivity to poisons increases with Topo I overexpression [104,105], while resistance to poisons is most commonly caused by a decrease in Topo I activity [50,106]. Camptothecin and its derivatives (topotecan, 10-hydroxy camptothecin, irinotecan, and rubitecan) stabilize the covalent Topo I-DNA cleavage complex by preventing DNA religation. Hence, they inhibit DNA relaxation and induce cleavage complexes (protein-linked DNA single-strand breaks) [103]. Since Topo I is the primary target of camptothecin, yeast cells with the Topo I gene deleted are resistant to this inhibitor. On the other hand, the expression of wild-type Topo I restores sensitivity to camptothecin [104,105]. In eukaryotic cells with high levels of resistance to camptothecin, mutations render the enzyme insensitive to the drug [107].

According to recent studies, the molecular mechanism of inhibition appears to be uncompetitive, considering that camptothecin does not bind to either the enzyme or the DNA substrate. Rather, it interacts with the enzyme–DNA complex to form a reversible nonproductive complex with Topo I [108].

**Figure 6:**
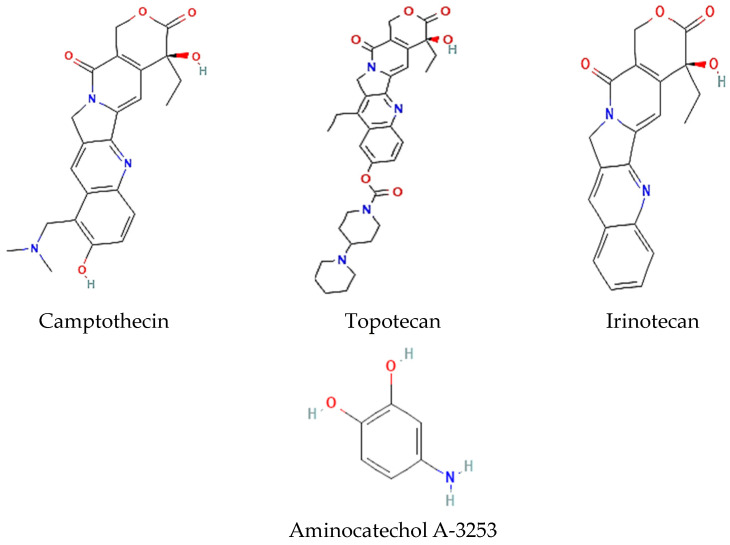
Chemical structure of selected inhibitors of Topo I.

Topotecan and irinotecan, two clinical drugs, are semisynthetic, water-soluble derivatives of camptothecin (the alkaloid plant compound) (Figure 6). Topotecan acts by forming a stable covalent complex with the DNA/Topo I aggregate, the so-called cleavable complex responsible for the cytotoxic properties of the drug [109]. Even though Topo I expression is independent of the cell cycle, the inhibition of Topo I through topotecan is most effective in the S phase [110]. Due to the formation of a stable cleavable complex, the DNA relaxation and subsequent replication are locked, resulting in the specific inhibition of Topo I. This has several implications for the biology of the cell. Firstly, S phase cells are irreversibly damaged by the irreversible strand break and the subsequent blocking of DNA synthesis and transcription. Furthermore, the replication forks are destroyed. Such irreversible damage leads to apoptosis and cell death [111].

**Figure 7:**
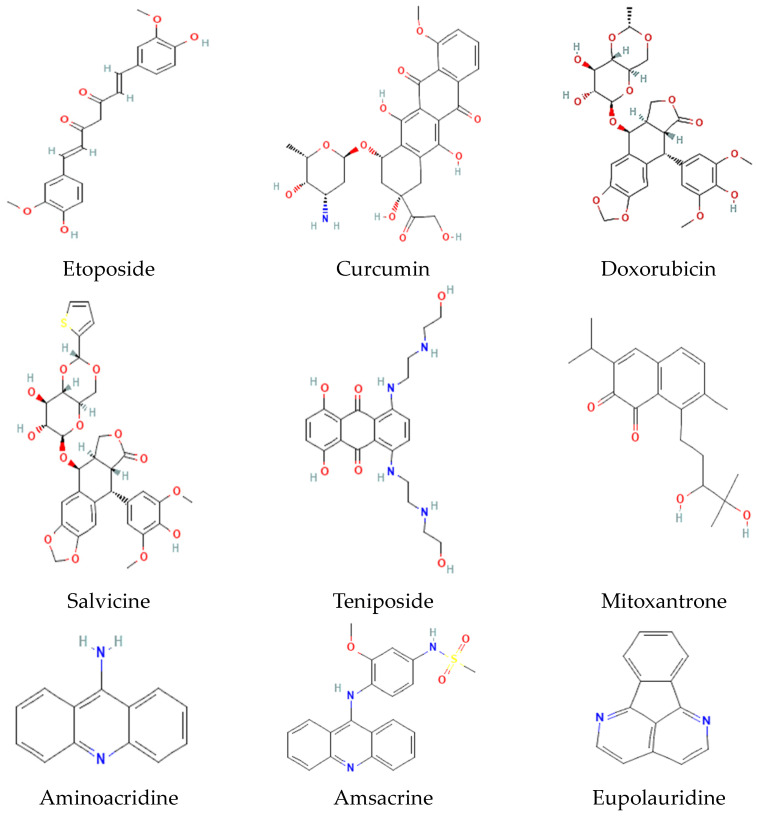
Chemical structure of selected inhibitors of Topo II.

Like Topo I inhibitors, Topo II inhibitors (Figure 7) are classified as poisons and catalytic inhibitors. The former encompasses the majority of clinical antitumor agents (etoposide, doxorubicin, mitoxantrone, salvicine, and teniposide) and represents frontline therapies for a wide spectrum of solid and hematological malignancies. They kill cancer cells by increasing the level of covalent Topo II-DNA complexes and preventing the cleaved DNA strand(s) from religation, thus causing the accumulation of double-strand breaks. The ‘classic’ Topo II poisons are characterized by their ability to induce DNA double-strand breakage through the stabilization of covalent complexes between the enzyme and DNA (cleavable complexes). This stabilization gives rise to mutations and eventually cell death. Some of the most relevant antitumor drugs that target and poison Topo II are anthracyclines (doxorubicin), epipodophyllotoxins (etoposide and teniposide), anthracenediones (mitoxantrone), and aminoacridines [112].

In the last few years, a novel group of drugs of diverse chemical nature has been reported as being non-classic ‘true’ catalytic inhibitors of mammalian Topo II. Unlike Topo II poisons, they lack the ability to stabilize the cleavable complex [113]. Hence, doses capable of efficiently inducing endoreduplication can be administered without provoking high levels of DNA and chromosome damage [114]. Additionally, they inhibit various genetic processes that involve the enzyme, such as DNA replication [115]. These drugs purportedly encompass aclarubicin, chloroquine, and the bisdioxopiperazines. However, whether the latter behave as Topo II poisons or inhibitors has been questioned. Catalytic inhibitors target the nuclear enzyme within the cell and interfere with various fundamental genetic processes (e.g., replication and transcription) and chromosome dynamics.

Based on distinct DNA binding patterns, Topo II poisons can be further divided into DNA intercalating and DNA non-intercalating agents. The latter (e.g., etoposide (1), teniposide (2), and quinolone) has relatively weak interactions with DNA and exert their function by trapping Topo II-DNA complexes. In contrast, DNA intercalating agents, such as doxorubicin, amsacrine, and mitoxantrone (which usually have coplanar aromatic frameworks), reversibly insert themselves into the base pairs of DNA and disrupt enzymes involved in DNA transcription and replication [116].

On the other hand, Topo II catalytic inhibitors are thought to kill tumor cells by inhibiting the essential enzymatic activity of the enzyme, thus impeding the conjunction of Topo II with DNA. They do not generate higher levels of Topo II covalent complexes, block the enzyme ATP-binding site (as do purine analogues), prevent the cleavage of DNA (as does merbarone), or inhibit the hydrolysis of ATP (as do bisdioxipiperazine analogues) [117]. The primary mechanism of action of etoposide and teniposide is the inhibition of the catalytic activity of eukaryote Topo II and the consequent stabilization of the normally transient covalent intermediate formed between the DNA substrate and the enzyme. Although teniposide is not substantially more potent than etoposide in terms of catalytic inhibition or stabilization of the DNA–enzyme intermediate, it is more readily taken up by cells. Hence, there is a greater accumulation of teniposide within the cells, causing a more robust cytotoxic capacity [79].

## 10. Selected Inhibitors of Topo I and II as Antifungal Agents

The first study to propose DNA topoisomerase inhibitors as antifungal agents dates back 30 years. The investigation was conducted on *C. albicans* and *A. niger* and supports the hypothesis that topoisomerase enzymes can be key targets for new antifungal agents [117]. According to another publication on camptothecin derivatives (selective inhibitors of the Topo I enzyme), certain structural modifications in the lead compound have significant effects on fungal cells. In addition, some of the derivatives display synergistic antifungal activity in combination with amphotericin B (a reference antifungal drug) [79]. As a result of these pioneering efforts, DNA topoisomerase inhibitors began to be considered as an alternative target for antifungal treatment.

In the 1990s, yeast strains constructed with mutations in the Topo II enzyme were reported to have resistance to Topo II inhibitors (etoposide and amsacrine) and high sensitivity to a Topo I inhibitor (camptothecin) [118]. Subsequent research discovered the bisdioxopiperazine-induced inhibition of Topo II of *S. cerevisiae* [119]. Moreover, different analogues of the antitumor drug rebeccamycin have exhibited activity against Topo I but not Topo II of *C. albicans* [120]. Eupolauridine proved to be capable of stabilizing the cleavage complex formed by the DNA topoisomerases of *C. albicans* and humans. Since topoisomerases have a greater response in humans, they are utilized as targets for the discovery of antifungal drugs [81,121].

Eupolauridine has been proven to completely inhibit the DNA relaxation activity of fungal Topo I of *S. cerevisiae* and other yeast strains but does not stabilize the cleavage complex [122]. Such stabilization is the mechanism of action of camptothecin, a selective Topo I inhibitor. To examine whether the inhibition of Topo I is the main mode of antifungal activity of eupolauridine, the authors used strains of *S. cerevisiae* with alterations in the Topo I gene. In the absence of Topo I, the antifungal activity of eupolauridine did not decrease. Rather, cells that lacked the enzyme were more sensitive to the drug, and the activity of eupolauridine was more evident in cells overexpressing Topo II. Thus, this enzyme is its actual target [122]. New analogues of eupolauridine were synthesized and tested, displaying activity against *C. albicans* and *C. neoformans* [123].

Mutations in Topo I have induced resistance to camptothecin, while mutations in Topo II have generated strong resistance to amsacrine and etoposide, two inhibitors of the enzyme [124]. Therefore, such drugs act on the DNA topoisomerases of yeast cells [125,126,127]. The in vitro evaluation of Topo II inhibitors of *C. albicans* has been facilitated by the cloning, expression, and characterization of the Topo II gene of yeast. The model is highly sensitive to the effects of amsacrine and doxorubicin [128].

Aminocatechol A-3253, a Topo I inhibitor, has activity against several pathogenic fungi, including *C. albicans* and *A. niger*. It produces a stronger effect on the Topo I of *Candida* than the same enzyme isolated from human cells [129].

As can be appreciated, *C. albicans* has been the *Candida* species most commonly chosen to test DNA topoisomerase inhibitors as an antifungal alternative. In 2010, the minimum inhibitory concentrations (MICs) of 10 inhibitors (of Topo I and II and other yeast enzymes) were determined in clinical isolates of *C. albicans*. Since growth and morphology were affected, the inhibitors were proposed as antifungal agents to treat *C. albicans* infections [130]. Two years later, eight inhibitors of Topo I and II were assessed on the growth of *A. fumigatus*, *A. niger*, *C. glabrata*, and *C. neoformans* [131]. They proved to be promising lead compounds for the development of new antifungal agents.

The most recent findings on Topo I and II inhibitors show that they reduce the growth, viability, and toxicity of *C. dubliniensis* and *C. glabrata* and act synergistically in the presence of fluconazole. Thus, *Candida* species are a good model for further research on topoisomerase inhibitors [47,132], and Topo II is a useful molecular target for the discovery of new drugs. Additionally, it is important to examine possible differences in the effects exerted by known inhibitors on fungal and human Topo II enzymes [48].

**Figure 8:**
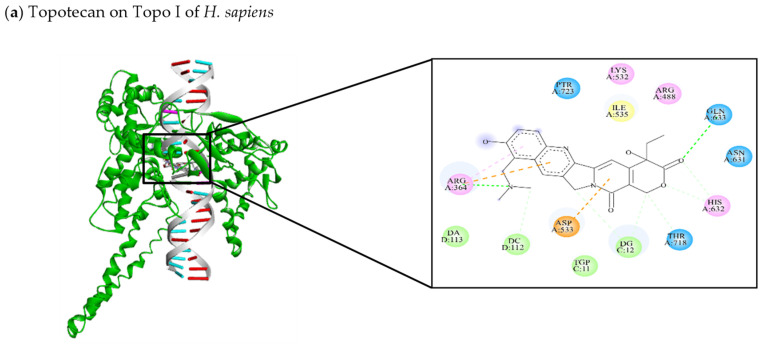

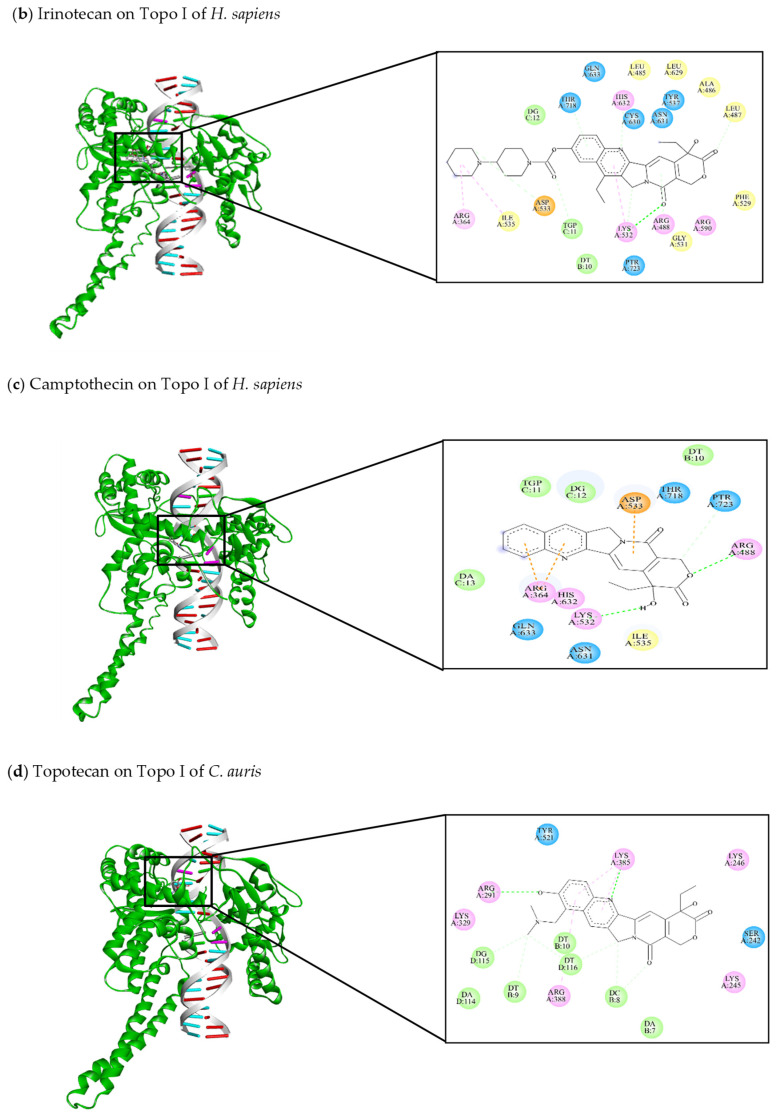

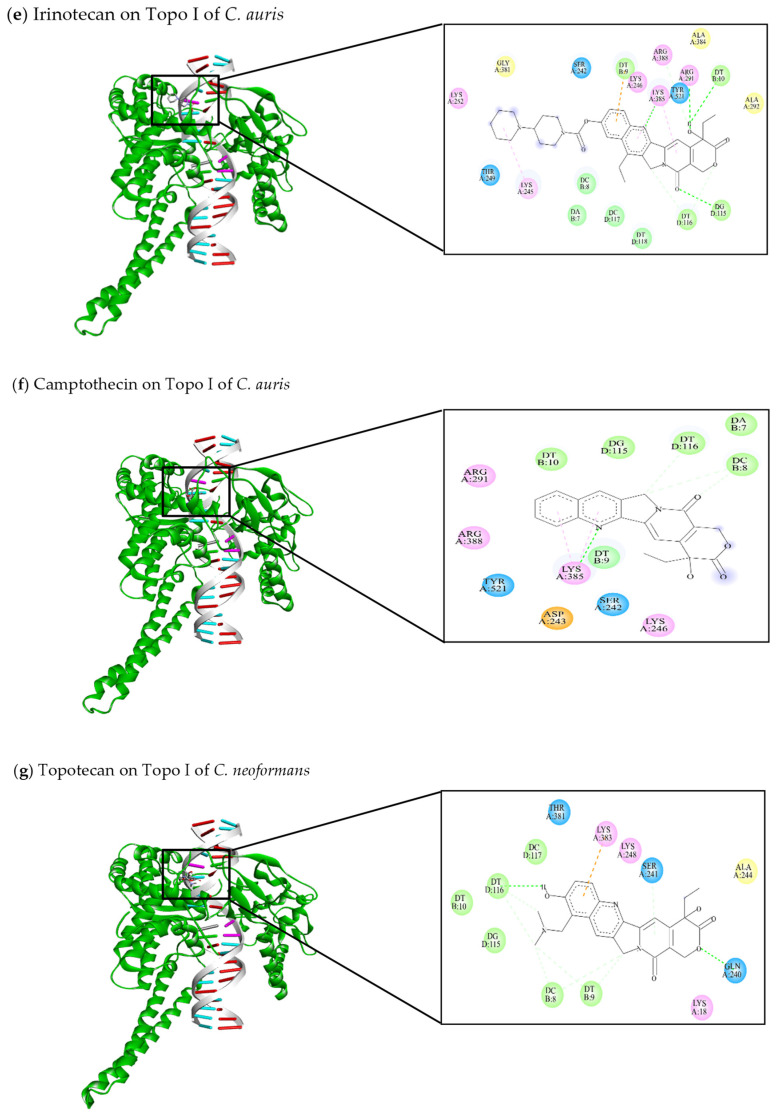

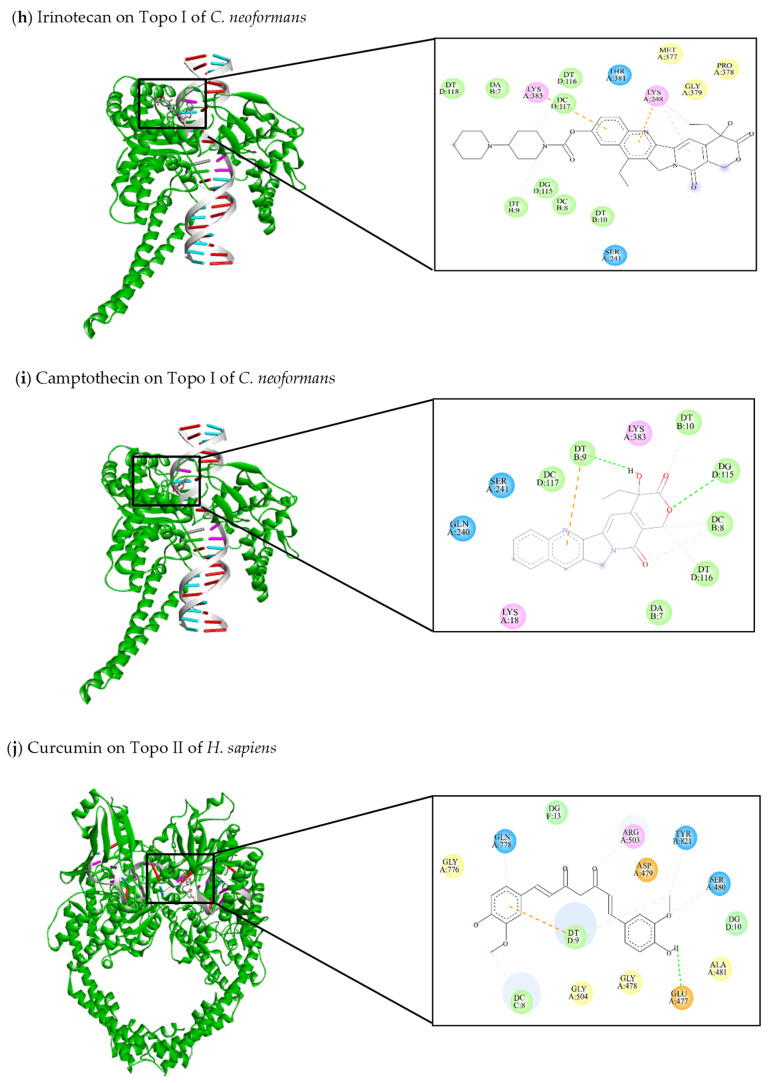

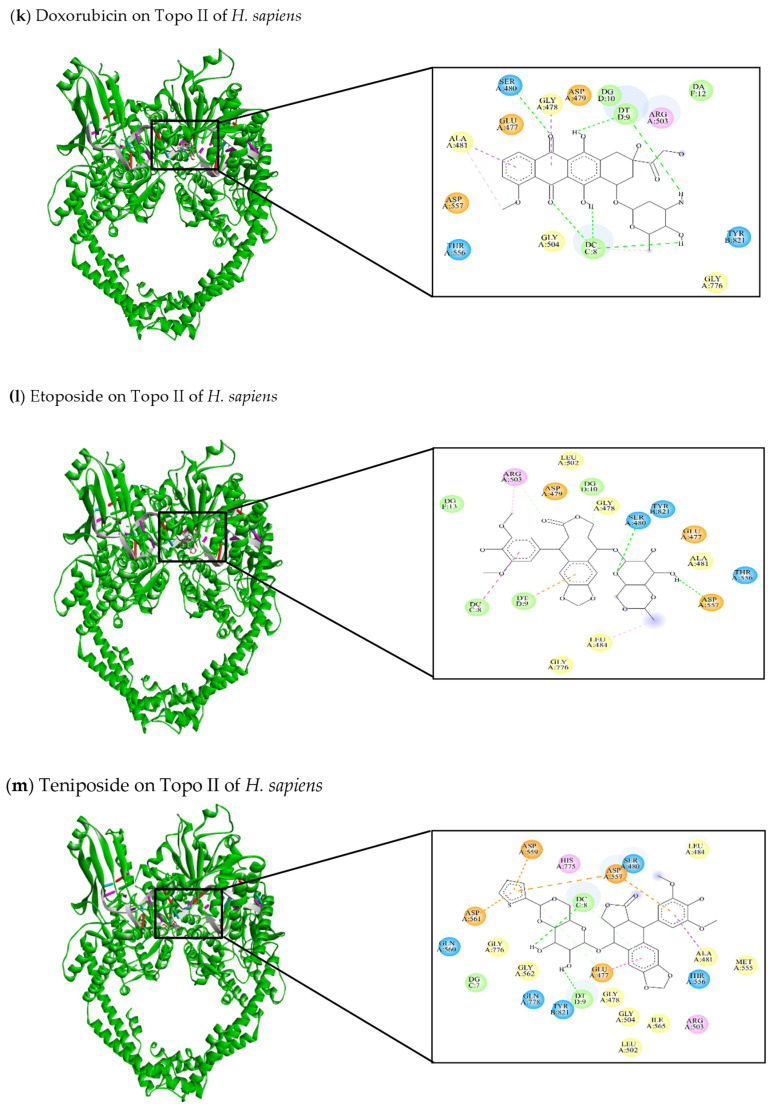

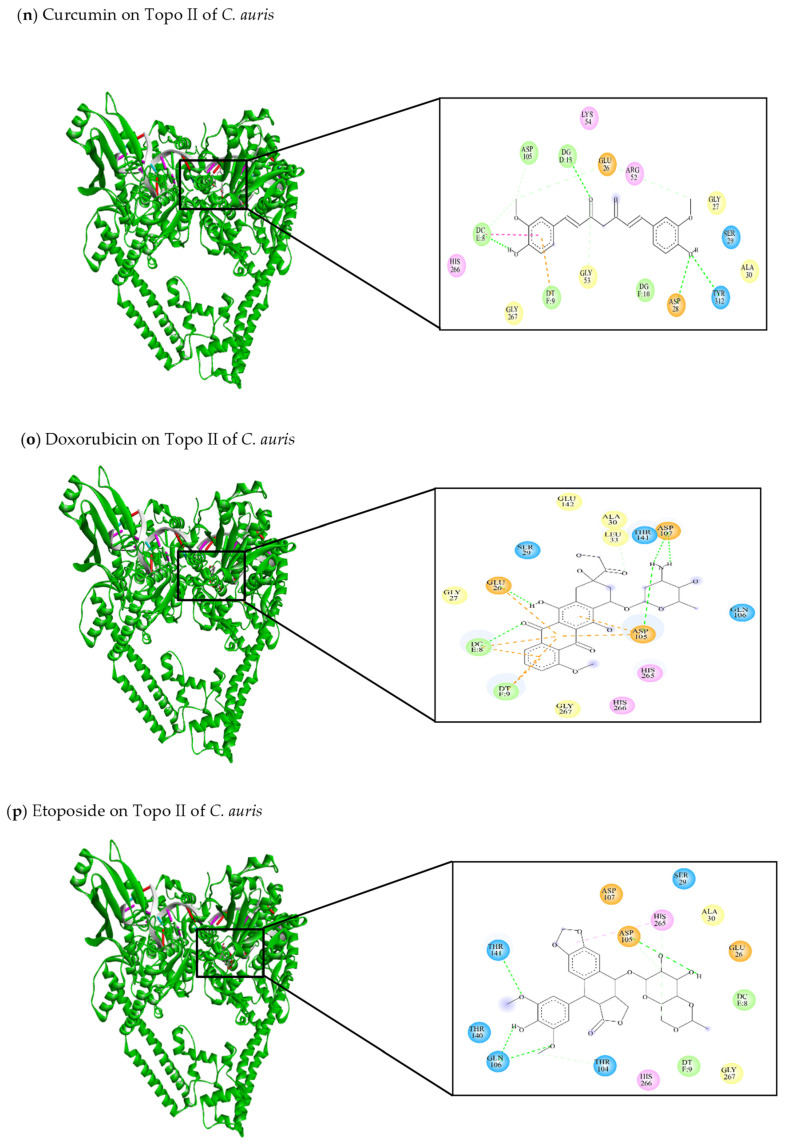

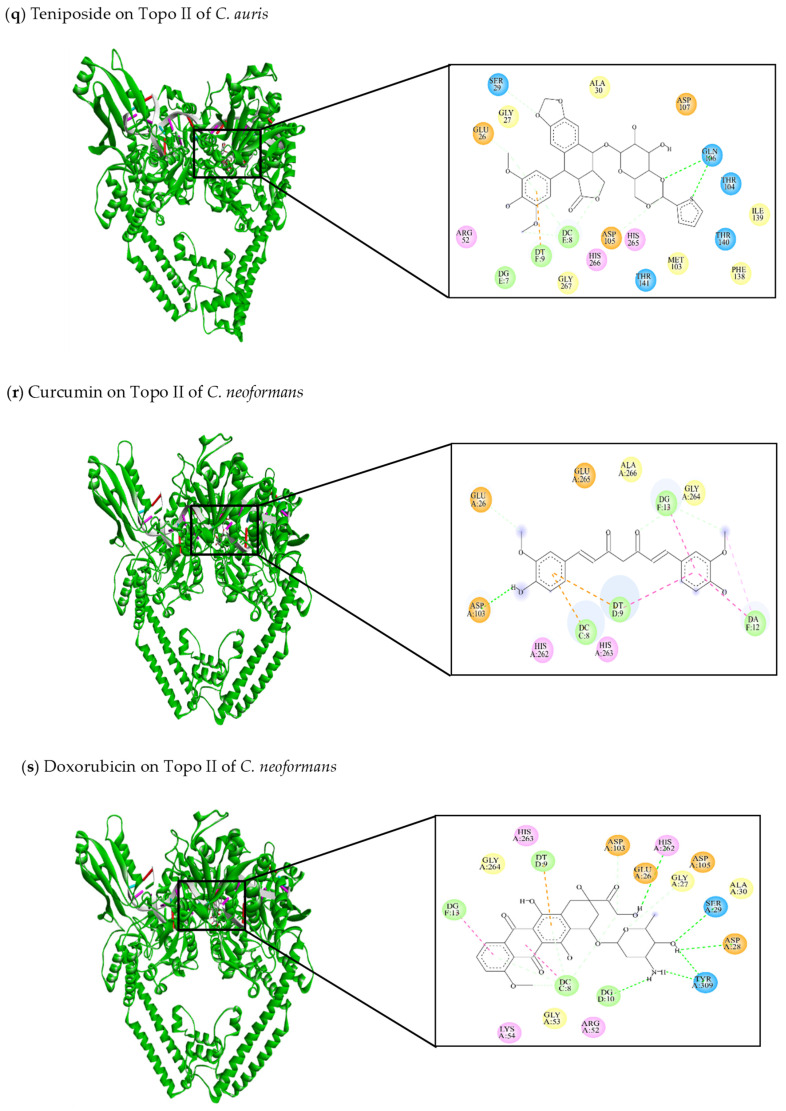

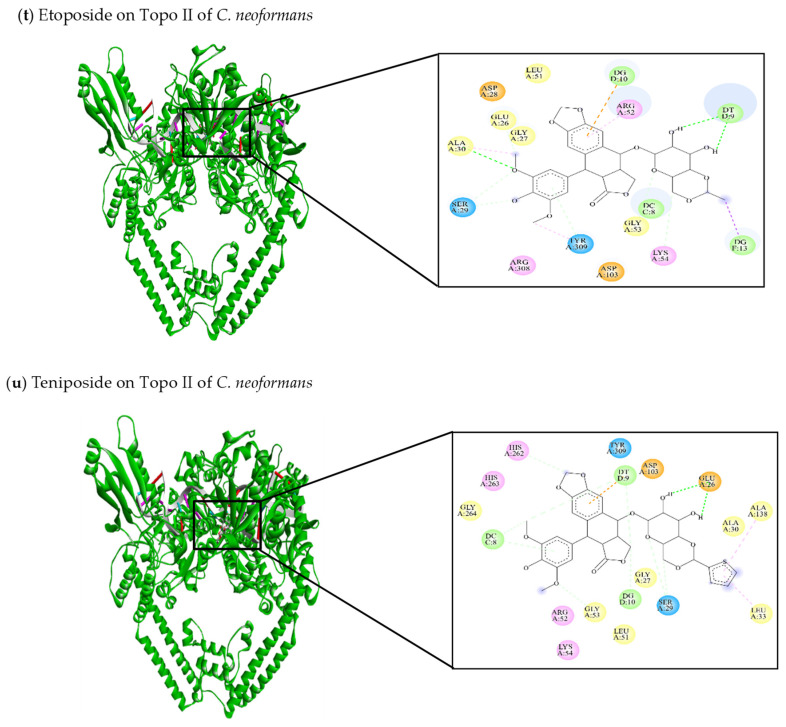
Illustration of the interactions between known inhibitors and Topo I or Topo II of a representative mammal (*H. sapiens*), Ascomycete (*C. auris*), and Basidiomycete (*C. neoformans*) (**a**–**u**). The images on the left portray the binding of the inhibitor to the enzyme. On the right, the 2D models depict different types of bonds in dotted lines: conventional hydrogen (dark green), carbon hydrogen (light green), π-π stacked (fuchsia), π-alkyl (pink), π-sigma (purple), π-anion, and π-actin (orange). The amino acids are illustrated in circles: pink (basic), orange (acid), blue (polar), and yellow (non-polar).

Docking studies have analyzed the binding mode as well as the probable mechanism by which inhibitors and their analogues recognize amino acid residues of the active site of Topo I and II enzymes. DNA molecular docking studies have been employed to compare the effects of topoisomerase inhibitors when targeting enzymes in yeasts versus *H. sapiens* [48,133,134,135,136,137]. The interactions of known inhibitors of Topo I (topotecan, irinotecan, and camptothecin) or Topo II (etoposide, teniposide, curcumin, and doxorubicin) with the respective enzymes of *C. auris*, *C. neoformans*, and *H. sapiens* are illustrated in Figure 8a–u.

Molecular docking was carried out with the 3D models for Topo I and II enzymes constructed by utilizing the crystallized structure of the human protein as a template. Hence, water molecules were removed and hydrogen atoms and Kollman charges were added to this crystallized structure. The 2D structures of the DNA topoisomerase inhibitors were downloaded from the Zinc database (https://zinc.docking.org/) (accessed on 1 July 2024) and optimized with Gaussian 16W and Gaussian 6.0 software to find the minimum energy conformation. Docking was performed on Autodock version 4.0 [138]. The grid box dimensions for Topo I were 58 × 76 × 72 A˚, centered at X = 89.899, Y = −0.426, and Z = 1.754, with a 0.375 A˚ spacing. The grid box dimensions for Topo II were 106 × 72 × 80 A˚, centered at X = 29.958, Y = 99.966, and Z = 42.272, with a 0.375 A˚ spacing. Where applicable, twists, torsion angles, atomic partial charges, and nonpolar hydrogens were added to the ligands. The hybrid Lamarckian genetic algorithm (with predetermined parameters) was applied for minimization. Of a total of 100 conformations, the one with the lowest binding energy (kcal/mol) was adopted. The docking results were viewed and edited in Discovery Studio.

The binding energy values of the docking interactions (listed in Table 2, expressed in kcal/mol) indicate the affinity of a compound for the active site of an enzyme. More negative values evidence greater affinity. The docking study also reveals the DNA and amino acid residues of the enzyme involved in binding, the hydrophilic and hydrophobic interactions generated between the residues and inhibitors, and the probable mechanism of action of the inhibitors. There are no recent reviews comparing the topology and structural characteristics of human and fungal Topo I and II enzymes. This is the first review that encompasses the general aspects of both enzymes and the in vitro and in silico interactions with their respective inhibitors. The valuable information gathered herein points to the importance of these inhibitors as an alternative in therapy against opportunistic human pathogenic fungi.

The amino acid residues Arg364, Arg488, Lys532, Asp533, Ile535, Asn631, His632, Gln633, Thr718, and PTR723 and nucleotide residues TGP11 and DG12 are reported to be present in Topo I of *H. sapiens* (Figure 8). They are highly conserved in the interactions with inhibitors of the enzyme. As mentioned in the table, topotecan and irinotecan [102,139,140,141,142] show a hydrophilic interaction with Thr718 (C-C), while some of the three Topo I inhibitors (topotecan, irinotecan, and camptothecin) exhibit hydrophobic interactions with Arg364 (π-cation), Arg364 (π-alkyl), and Asp533 (π-anion). In the interactions between Topo I of *C. auris* and its inhibitors, various amino acid residues (Ser242, Lys245, Lys246, Arg291, Lys329, Lys385, Arg388, and Tyr521) and nucleotide residues (DA7, DC8, DT9, DT10, DG115, and DT116) are conserved. Hydrophilic interactions are predominant for Arg291 (C-HO) with topotecan and irinotecan and for Lys385 (C-HO) with all three inhibitors. On the other hand, there is a hydrophobic interaction between Lys385 (π-alkyl) and all three Topo I inhibitors. Regarding Topo I of *C. neoformans*, Ser241, Lys248, Thr381, and Lys383 participate in the interactions with the three inhibitors, and Lys18 and Gln240 are involved in binding with irinotecan and camptothecin [47,99,142,143,144]. Additionally, the nucleotide residues DC8, DT9, DT10, DC117, DG115, and DT116 participate in inhibitor binding. Nucleotide residues DT9 (C-C) and DT116 (C-C) play a role in the predominantly hydrophilic interactions with the three inhibitors, and the hydrophobic interaction of Lys383 (π-anion) with topotecan and irinotecan is conserved [99,139,140,141,142].

Regarding the Topo II enzyme of *H. sapiens*, the amino acid residues Glu477, Gly478, Asp479, Ser480, Ala481, Arg503, Gly504, Gly776, Gln778, and Tyr821 and nucleotide residues DC8 and DT9 are among those described in the literature as part of the active site (Figure 8) [47,144,145]. The hydrophilic interactions of Ser480 (C-HO) with curcumin and doxorubicin [47,144,145], and of Arg503 (C-C) with curcumin and etoposide [141,144,145,146], are conserved, while there is a hydrophobic interaction of Ala481 (π-sigma) with doxorubicin and teniposide [101,145,147]. In Topo II of *C. auris*, the amino acid residues Glu26, Gly27, Asp28, Ser29, Ala30, Arg52, Gly53, Lys54, Asp105, His266, Gly267, and Tyr312 and the nucleotide residues DC8 and DT9 are conserved. Hydrophilic interactions have been identified for Asp105 (C-HO) with doxorubicin and etoposide [14,15,16,17,18,19,20,21,22,23,24,25,26,27,28,29,30,31,32,33,34,35,36,37,38,39,40,41,42,43,44,45,46,47,48,49,50,51,52,53,54,55,56,57,58,59,60,61,62,63,64,65,66,67,68,69,70,71,72,73,74,75,76,77,78,79,80,81,82,83,84,85,86,87,88,89,90,91,92,93,94,95,96,97,98,99,100,101,102,103,104,105,106,107,108,109,110,111,112,113,114,115,116,117,118,119,120,121,122,123,124,125,126,127,128,129,130,131,132,133,134,135,136,137,138,139,140,141,142,143,144,145,146,147], Gln106 (C-HO) with etoposide and teniposide [145,146], and Glu26 (C-C) with curcumin and teniposide [144,146]. A hydrophobic interaction exists for DT9 (π-anion) with curcumin, doxorubicin, and teniposide. In relation to Topo II of *C. neoformans*, the amino acid residues Glu26, Asp103, His262, His263, Gly264, Glu265, and Ala266 and nucleotide residues DC8, DT9, DG10, and DG13 are conserved. The hydrophilic interaction of Glu26 with curcumin is of the C-C type, while with teniposide, it is of the C-HO type. Etoposide and teniposide retain the interaction with Ser29 (C-C). There is a hydrophobic π-anion interaction of DT9 with all three Topo II inhibitors. Although only some of the conserved interactions between residues and one, two, or more Topo I and II inhibitors are presently mentioned, all such amino acid and nucleotide residues are at the active site of the enzymes. Since the active sites of the enzymes are apparently conserved in relation to *H. sapiens* [144,145,146,147,148] and *Candida* species, known inhibitors of these enzymes in *H. sapiens* could possibly be used as antifungal agents.

**Table 2:** Results of the interactions between known inhibitors and the Topo I or Topo II enzymes of a representative mammal (*H. sapiens*), Ascomycete (*C. auris*), and Basidiomycete (*C. neoformans*).

Compound	Binding Energy (kcal/mol)	Residues Interacting with the Ligand	Nucleotide Residues	Polar Interactions	Hydrophobic Interactions
	**Topo I of *H. sapiens***				
[104,141,142,143] **Topotecan**	−9.42	Arg364, Arg488, Lys532, Asp533, Ile535, Asn631, His632, Gln633, Thr718, PTR723	TGP11, DG12, DC112, DA113	Arg364 (C-HO) Gln633 (C-HO) His632 (C-C) Thr718 (C-C) DG12 (C-C) DC112 (C-C)	Arg364 (π-cation) Arg364 (π-alkyl) Asp533 (π-anion)
[144] **Irinotecan**	−11.47	Arg364, Leu485, Ala486, Leu487, Arg488, Phe 529, Gly531, Lys532, Asp533, Ile535, Tyr537, Cys630, Leu629, Asn631, His632, Gln633, Thr718, PTR723	DT10, DG12, TGP11	Lys532 (C-HO) Lys532 (C-C) Asp533 (C-C) Cys630 (C-C) Thr718 (C-C) TGP11 (C-C)	Lys532 (π-alkyl) Arg364 (π-alkyl) Ile535 (π-alkyl)
[46,103,145,146] **Camptothecin**	−9.3	Arg364, Arg488, Lys532, Asp533, Ile535, Asn631, His632, Gln633, Thr718, PTR723	DT10, TGP11, DG12, DA13	Arg488 (C-HO) Lys532 (C-HO) PTR723 (C-C)	Arg364 (π-cation) Asp533 (π-anion)
	**Topo I of *C. auris***				
This review **Topotecan**	−9.08	Ser242, Lys245, Lys246, Arg291, Lys329, Lys385, Arg388, Tyr521	DA7, DC8, DT9, DT10, DA114, DG115, DT116	Arg291 (C-HO) Lys385 (C-HO) DC8 (C-C) DT9 (C-C) DG115 (C-C) DT116 (C-C)	Lys385 (π-alkyl) Arg388 (π-alkyl)
This review **Irinotecan**	−11.62	Ser242, Lys245, Lys246, Thr249, Lys252, Arg291, Ala292, Gly381, Ala384, Lys385, Arg388, Tyr521	DA7, DC8, DT9, DT10, DG115, DT116, DC117, DT118	Arg291 (C-HO) Lys385 (C-HO) DT10 (C-HO) DG115 (C-HO) DT116 (C-C)	Lys245 (π-alkyl) Arg291 (π-alkyl) DT9 (π-anion)
This review **Camptothecin**	−9.26	Ser242, Asp243, Lys246, Arg291, Lys385, Arg388, Tyr521	DA7, DC8, DT9, DT10, DG115, DT116	Lys385 (C-HO) DC8 (C-C) DT116 (C-C)	Lys385 (π-alkyl)
	**Topo I of *C. neoformans***				
This review **Topotecan**	−9.28	Lys18, Gln240, Ser241, Ala244, Lys248, Thr381, Lys383	DC8, DT9, DT10, DC117, DG115, DT116	Gln240 (C-HO) DC8 (C-C) DT9 (C-C) DT116 (C-C)	Lys383 (π-anion)
This review **Irinotecan**	−10.67	Ser241, Lys248, Met377, Pro378, Gly379, Thr381, Lys383	DA7, DC8, DT9, DT10, DG115, DT116, DC117, DT118	DT9 (C-C) DG115 (C-C) DT116 (C-C)	Lys248 (π-alkyl) Lys383 (π-anion)
This review **Camptothecin**	−8.4	Lys18, Gln240, Ser241, Lys383	DA7, DC8, DT9, DT10, DG115, DT116, DC117	DT9 (C-HO) DG115 (C-HO) DC8 (C-C) DT10 (C-C) DT116 (C-C)	DT9 (π-anion)
	**Topo II of *H. sapiens***				
[47,144] **Curcumin**	−8.73	Glu477, Gly478, Asp479, Ser480, Ala481, Arg503, Gly504, Gly776, Gln778, Tyr821	DC8, DT9, DG10, DG13	Glu477 (C-HO) Ser480 (C-C) Gln778 (C-C) Arg503 (C-C) Tyr821 (C-C) DT9 (C-C)	DT9 (π-anion)
[145] **Doxorubicin**	−9.67	Glu477, Gly478, Asp479, Ser480, Ala481, Arg503, Gly504, Thr556, Asp557, Gly776, Tyr821	DC8, DT9, DG10, DA12	Ser480 (C-HO) DC8 (C-HO) DT9 (C-HO)	Gly478 (π-sigma) Ala481 (π-Alkyl) Ala481 (π-sigma)
[47,141,145,146] **Etoposide**	−7.2	Glu477, Gly478, Asp479, Ser480, Ala481, Leu484, Leu502, Arg503, Thr556, Asp557, Gly776, Tyr821	DC8, DT9, DG10, DG13	Ser480 (C-HO) Asp557 (C-HO) Arg503 (C-C)	Leu484 (π-alkyl) Arg503 (π-alkyl) DC8 (π-alkyl) DT9 (π-anion)
[103,146] **Teniposide**	−7.0	Glu477, Gly478, Ser480, Ala481, Leu484, Leu502, Arg503, Gly504, Met555, Thr556, Asp557, Asp559, Gln560, Asp561, Gly562, Ile565, His775, Gly776, Gln778, Tyr821	DG7, DC8, DT9	Glu477 (C-C) DC8 (C-HO) DT9 (C-HO)	Ala481 (π-sigma) Glu477 (π-π stacked) Asp557 (π-anion) Asp559 (π-anion) Asp561 (π-anion)
	**Topo II of *C. auris***				
This review **Curcumin**	−8.27	Glu26, Gly27, Asp28, Ser29, Ala30, Arg52, Gly53, Lys54, Asp105, His266, Gly267, Tyr312	DC8, DT9, DG10, DG13	Glu26 (C-C) Asp28 (C-HO) Gly53 (C-C) Asp105 (C-C) Tyr312 (C-HO) DC8 (C-HO) DG13 (C-HO)	DC8 (π-π stacked) DT9 (π-anion)
This review **Doxorubicin**	−9.53	Glu26, Gly27, Ser29, Ala30, Leu33, Asp105, Gln106, Asp107, Thr141, Glu142, His265, His266, Gly267	DC8, DT9	Glu26 (C-HO) Asp105 (C-HO) Asp107 (C-HO) Leu33 (C-C)	Glu26 (π-anion) Asp105 (π-anion) Asp107 (π-anion) DC8 (π-anion) DT9 (π-anion)
This review **Etoposide**	−7.48	Glu26, Ser29, Ala30, Thr104, Asp105, Gln106, Asp107, Thr140, Thr141, His265, His266, Gly267	DC8, DT9	Thr104 (C-C) Asp105 (C-HO) Gln106 (C-HO) Thr141 (C-HO)	His265 (π-alkyl)
This review **Teniposide**	−8.54	Glu26, Gly27, Ser29, Ala30, Arg52, Met103, Thr104, Asp105, Gln106, Asp107, Phe138, Ile139, Thr140, Thr141, His265, His266, Gly267	DG7, DC8, DT9	Glu26 (C-C) Ser29 (C-C) Gln106 (C-HO) DC8 (C-C)	DT9 (π-anion)
	**Topo II of *C. neoformans***				
This review **Curcumin**	−8.4	Glu26, Asp103, His262, His263, Gly264, Glu265, Ala266	DC8, DT9, DA12, DG13	Glu26 (C-C) Asp103 (C-HO) DG13 (C-C)	DC8 (π-anion) DT9 (π-anion) DA12 (π-alkyl) DA12 (π-π stacked) DT9 (π-π stacked)
This review **Doxorubicin**	−11.04	Glu26, Gly27, Asp28, Ser29, Ala 30, Arg52, Gly53, Lys54, Asp103, Asp105, His262, His263, Gly264, Tyr309	DC8, DT9, DG10, DG13	Gly27 (C-C) Asp28 (C-HO) Ser29 (C-HO) Asp103 (C-C) His262 (C-HO) Tyr309 (C-HO) DC8 (C-C)	DC8 (π-alkyl) DT9 (π-anion) DG13 (π-alkyl)
This review **Etoposide**	−9.4	Glu26, Gly27, Asp28, Ser29, Ala30, Leu51, Arg52, Gly53, Lys54, Asp103, Arg308, Tyr309	DC8, DT9, DG10, DG13	Ser29 (C-C) Ala30 (C-HO) Tyr309 (C-C) DC8 (C-C) DT9 (C-HO)	Ala30 (π-alkyl) Arg52 (π-alkyl) Tyr309 (π-alkyl) DG13 (π-sigma)
This review **Teniposide**	−10.58	Glu26, Gly27, Ser29, Ala30, Leu33, Leu51, Arg52, Gly53, Lys54, Asp103, Ala138, His262, His263, Gly264, Tyr309	DC8, DT9, DG10	Glu26 (C-HO) Ser29 (C-C) Gly53 (C-C) His262 (C-C) DC8 (C-C) DG10 (C-C)	Leu33 (π-alkyl) Ala138 (π-alkyl) DT9 (π-anion)

According to the interactions observed in the molecular docking results, we can observe that, in all cases, the human topoisomerase I inhibitors showed electrostatic interactions of type π-anion with Asp533, and this interaction takes place between the electron-deficient aromatic ring of pyridone of topotecan or camptothecin and the carboxylate group rich in electron density. This interaction was not observed in the case of topoisomerase I of *C. auris* and *C. neoformans*; therefore, the introduction of an electron-withdrawing group (for example, NO_2_, CN, Cl, F, Br) in the pyridone ring of Topo I inhibitors would increase the interaction with Asp33 and, in general, with acidic amino acids and would make these compounds more selective towards human topoisomerase I enzyme. Regarding the topoisomerase II enzyme, although curcumin and doxorubicin do not have structural similarity with etoposide and teniposide, it is evident that all these inhibitors showed a greater number of interactions with basic amino acids at the active site of *C. auris* and *C. neoformans* compared to what was observed in *H. sapiens*. It is well known that electron-donating groups, such as OMe, OH, NH_2_, and SH in aromatic rings, which increase the electron density, favor the interaction with basic amino acids due to the latter having cationic groups and can experience electrostatic interactions. Therefore, the introduction of additional electron-donating groups in these drugs would make these inhibitors more selective towards topo II of *C. auris* and *C. neoformans*.

## 11. Therapeutic Potential of Topo I and II Inhibitors in Fungal Organisms

Reports in the literature have clearly pointed to the therapeutic potential of using known and proposed Topo I and II inhibitors for fungal infections. For example, acridine thiosemicarbazide derivatives exhibit antifungal activity with an MIC of 10–80 μM. In recent studies, researchers have obtained a selective derivative of acridine, named M14, with anticandidal and anti-dermatophyte activity. This compound inhibits the growth of all reference and clinical strains of *Candida* and dermatophytes, with an MIC ranging from 7.81 to 31.25 μg/mL. The presence of this acridine derivative prevents *C. albicans* biofilm formation and reduces the viability of preformed biofilms at concentrations lower than the MIC [148]. Microscopic evaluation of the hyphal growth of *C. albicans* in the presence of M14 evidences the complete inhibition of the yeast-to-mycelia transformation. Similarly, severe inhibition of the hyphal growth of *Trichophyton rubrum* has been reported [149].

Aclarubicin and idarubicin are effective against *Aspergillus niger*, *C. glabrata*, *C. albicans*, and *C. neoformans*, with MICs ranging from 1.8 to 8.4 μg/mL. The results of the viability assay indicate a fungistatic mode of action of aclarubicin [132,133].

Camptothecin and etoposide inhibit the growth of the *C. albicans* ATTC 18804 strain at an MIC of 50 μg/mL [132], while derivatives of camptothecin (topotecan and irinotecan) inhibit the growth of *C. glabrata*, *A. fumigatus*, *A. niger*, and *C. neoformans* at an MIC of 42.1 and 57.8 μg/mL, respectively [133]. Camptothecin and etoposide are capable of inhibiting the *C. glabrata* CBS138 strain at an MIC of 2.5 and 5 μg/mL, respectively. Camptothecin, curcumin, and etoposide inhibit *C. dubliniensis* at MICs ranging from 0.078 to 0.312 μg/mL.

As can be appreciated, the reported compounds or their analogues could possibly be used against a wide variety of fungal infections.

## 12. Future Perspectives on Topo I and II Enzymes as Alternative Targets for Antifungal Drugs

Given the increasing prevalence of fungal infections among immunocompromised patients in hospitals, the limited options for antifungal therapy, and the sharp rise in the development of multi-drug-resistant strains of pathogenic fungi, there is an urgent need to discover new drugs with novel therapeutic targets. Type I and II DNA topoisomerase enzymes in fungal organisms represent a plausible alternative target due to their structural characteristics. Topoisomerase inhibitors have been shown to effectively decrease yeast growth, and topoisomerase enzymes have served as a model to evaluate new inhibitors. Taking known inhibitors as lead compounds, analogues can be designed and synthesized with structural modifications aimed at attaining improved efficacy on a wide variety of fungal organisms and reduced toxicity. In silico studies such as molecular dynamics carried out with a biological matrix provide valuable clues about the behavior of a Topo I or II inhibitor when binding at the active site of the enzyme. Future research is needed to elaborate recombinant proteins of Topo I and II enzymes of fungal organisms in order to employ them as study models for examining the inhibitory activity of new antifungal compounds. Additionally, the generation of point mutants at the active site of the enzymes will help to reveal the role of certain amino acid residues in the activity of proposed inhibitors. Finally, it is important to continue the search for new strategies to synthesize possible Topo I and II inhibitors.

## 13. Conclusions

DNA topoisomerase enzymes are the molecular targets of some anticancer agents and antimicrobials. Although they were suggested as antifungal targets in the 1990s, very little research has focused on the structural differences between their fungal and human forms. Because of the scarcity of in silico studies, however, little is known about their binding mode and probable mechanism of action. The present review summarizes the available information on the biology, topology, structural characteristics, DNA sequences, and genes of Topo I and II enzymes. A close phylogenetic relationship was found between the enzymes of the thirty-two organisms herein analyzed. A comparison was made between the way Topo I and Topo II inhibitors bind to the amino acid and DNA residues of the active site of the enzymes. Topo I and II enzymes have proven to be valuable models for testing proposed antifungal agents aimed at combating a wide spectrum of pathogenic fungi. According to the results of molecular docking, irinotecan and doxorubicin presented the best binding energy values, which makes them worthy of further research to develop derivatives with greater antifungal potential. However, it is necessary to validate the findings of molecular docking with molecular dynamics.

## Figures and Tables

**Table 1:** Details of the sequences of Topo I and Topo II enzymes from mammals and fungi.

Organism	Accession Number	Family	Gene Length (bp)	Protein Size (aa)	Accession Number	Family	Gene Length (bp)	Protein Size (aa)
***Homo sapiens***	NP_003277	Topo I	3734	765	NP_001058	Topo II	5695	1531
***Mus musculus***	NP_033434	Topo I	3859	767	XP_006533217	Topo II	5233	1527
***Rattus norvegicus***	NP_072137	Topo I	2304	767	P41516	Topo II	4581	1526
***Saccharomyces cerevisiae***	NP_014637	Topo I	2310	769	AAM005481	Topo II	4287	1428
***Schizosaccharomyces pombe***	NP_596209	Topo I	2761	814	NP_595805	Topo II	6626	1485
***Yarrowia lipolytica***	KAB8283935	Topo I	2283	760	RDW22562	Topo II	6513	1537
***Candida albicans***	XP_714305	Topo I	2343	780	KHC76731	Topo II	4386	1461
***Candida auris***	PSK79030	Topo I	2250	749	PSK77735	Topo II	4377	1458
***Candida haemulonii***	XP_025340562	Topo I	2250	749	XP_025343359	Topo II	4377	1458
***Clavispora lusitaniae***	OVF09121	Topo I	2295	764	OVF06965	Topo II	4230	1409
***Candida dubliniensis***	XP_002420888	Topo I	2334	777	XP_002420251	Topo II	4386	1461
***Candida tropicalis***	XP_002548660	Topo I	2334	777	XP_002549527	Topo II	4197	1398
***Candida krusei***	AWT08596	Topo I	2187	728	XP_029322551	Topo II	4272	1423
***Kluyveromyces marxianus***	BAP69451	Topo I	2304	767	XP_022678261	Topo II	4350	1449
***Candida parapsilosis***	KAF6043449	Topo I	2466	821	CCE43879	Topo II	4332	1443
***Candida orthopsilosis***	XP_003870756	Topo I	2418	805	XP_003867280	Topo II	4422	1473
***Candida glabrata***	XP_445795	Topo I	2154	717	KTB13451	Topo II	4221	1406
***Candida utilis***	XP_020072437	Topo I	2772	755	XP_020068929	Topo II	4324	1416
***Meyerozyma guilliermondii***	EDK40177	Topo I	2265	754	XP_001483297	Topo II	4203	1400
***Ustilago maydis***	XP_011388171	Topo I	3060	1019	XP_011389948	Topo II	4557	1518
***Coccidioides immitis***	XP_001245381	Topo I	3335	905	TPX24629	Topo II	5157	1718
***Aspergillus niger***	GAQ33639	Topo I	2595	864	XP_001392968	Topo II	5268	1707
***Aspergillus clavatus***	XP_001269805	Topo I	2661	886	XP_001268423	Topo II	5208	1735
***Aspergillus nidulans***	AAO19447	Topo I	2990	871	XP_663010	Topo II	5130	1709
***Aspergillus terreus***	GES60315	Topo I	2643	880	XP_001212600	Topo II	5199	1732
***Aspergillus fumigatus***	KAF4263330	Topo I	2604	867	XP_751245	Topo II	4983	1660
***Penicillium marneffei***	KFX43083	Topo I	2583	860	XP_002145834	Topo II	5216	1696
***Paracoccidioides brasiliensis***	ODH53586	Topo I	2718	905	XP_010761698	Topo II	5850	1949
***Fusarium oxysporum***	XP_031047401	Topo I	4701	909	EWZ41880	Topo II	5214	1737
***Blastomyces dermatitidis***	EEQ86368	Topo I	2736	911	EGE78098	Topo II	5415	1804
***Histoplasma capsulatum***	EEH11244	Topo I	2730	909	XP_001536323	Topo II	5427	1808
***Cryptococcus neoformans var. neoformans***	XP_572925	Topo I	2953	926	XP_566700	Topo II	4182	1272

## Supplementary Materials

The following supporting information can be downloaded at: https://www.mdpi.com/article/10.3390/jof10090629/s1, Figure S1: Alignment of the amino acid sequences of the active site region of the DNA topoisomerase I and II enzymes of the families of organisms under study; Figure S2: Overlapping of the 3D models from Topo I (a) and Topo II (b) of 29 fungal and 3 mammalian organisms (including *H. sapiens*); Figure S3: Ramachandran plot of the models of Topo I (a,b) and Topo II (c,d) from representative organism (*C. auris* and *C. neoformans*, respectively); Table S1: Analysis of the identity and similarity of Topo I enzymes from mammals and fungi; Table S2: Analysis of the identity and similarity of Topo II enzymes from mammals and fungi; Table S3: RMSD value of Topo I and II 3D models of representative organisms.
