# Supplementary material for: Review and Current Perspectives on DNA Topoisomerase I and II Enzymes of Fungi as Study Models for the Development of New Antifungal Drugs
Source: J Fungi (Basel). 2024 Sep 3;10(9):629. doi: 10.3390/jof10090629 (PMC11432948; doi:10.3390/jof10090629)
Supplement: Supplementary file 1 [file jof-10-00629-s001.zip › jof-3152591-supplementary.pdf]

Supplementary Material

(**a**)

Ustilago maydis	NTSDVALSTSKLNYIDPRI
Homo sapiens	ENKQIALGTSKLNYLDPRI
Mus musculus	ENKQIALGTSKLNYLDPRI
Rattus norvegicus	ENKQIALGTSKLNYLDPRI
Cryptococcus neoformans var. neoformans	KLKDVALGTSKINYIDPRL
Schizosaccharomyces pombe	ENKTTALGTSKINYIDPRL
Yarrowia lipolytica	DNSSVALGTSKMNYIDPRL
Candida krusei	ENSTVSLGTSKMNYIDPRL
Kluyveromyces marxianus	DNSTVALGTSKINYIDPRL
Saccharomyces cerevisiae	ENSQVSLGTSKINYIDPRL
Candida glabrata	ANSEVSLGTSKINYIDPRL
Candida utilis	DNSQVALSTSKINYIDPRL
Clavispora lusitaniae	DNSEVSLGTSKLNYIDPRL
Candida auris	DNSEVSLGTSKMNYIDPRL
Candida haemulonii	DNSEVSLGTSKLNYIDPRL
Meyerozyma guilliermondii	DNSEVSLGTSKLNYIDPRL
Candida parapsilosis	DNSEVSLGTSKMNYIDPRL
Candida orthopsilosis	DNSEVSLGTSKMNYIDPRL
Candida tropicalis	DNSEVSLGTSKMNYIDPRL
Candida albicans	DNSEVSLGTSKMNYIDPRL
Candida dubliniensis	DNSEVSLGTSKMNYIDPRL
Fusarium oxysporum	GNKEVALGTSKINYIDPRL
Coccidioides immitis	DNKEVALGTSKINYIDPRL
Paracoccidioides brasiliensis	NNKEVALGTSKINYIDPRL
Blastomyces dermatitidis	NNKEVALGTSKINYIDPRL
Histoplasma capsulatum	NNKEVALGTSKINYIDPRL
Penicillium marneffei	NNKEVALGTSKINYIDPRL
Aspergillus clavatus	DNKEVALGTSKINYIDPRL
Aspergillus fumigatus	DNKEVALGTSKINYIDPRL
Aspergillus niger	ENKEVALGTSKINYIDPRL
Aspergillus nidulans	DNKEVALGTSKINYIDPRL
Aspergillus terreus	DNKEVALGTSKINYIDPR

(**b**)

Homo sapiens	CTLILTEGDSAKTLAVSGLGVVGRDKYGVFPLRGKILNVREASHKQIMENAEINN
Mus musculus	CTLILTEGDSAKTLAVSGLGVVGRDKYGVFPLRGKILNVREASHKQIMENAEINN
Rattus norvegicus	CTLILTEGDSAKTLAVSGLGVVGRDKYGVFPLRGKILNVREASHKQIMENAEINN
Yarrowia lipolytica	CTLILTEGDSAMTMANAGLSVIGRERYGAFPLRGKMLNVREASTDQIMKNAEIQA
Candida utilis	CTLILTEGDSALALAVAGLAVVGRDYYGCFPLRGKPLNVREATASQITNNAEINA
Candida krusei	CTLILTEGDSAKSLAVQGLAVVGRNYYGCFPLRGKVLNVREASTDQIMKNGAIQA
Kluyveromyces marxianus	CTLVLTEGDSALSLAVAGLAVVGRDYYGCFPLRGKMLNVREATPDQITKNAEIQA
Saccharomyces cerevisiae	CTLVLTEGDSALSLAVAGLAVVGRDYYGCYPLRGKMLNVREASADQILKNAEIQA
Candida glabrata	CTLILTEGDSALSLAVAGLAVVGRDYYGCYPLRGKMLNVREATADQILKNAEIQA
Clavispora lusitaniae	CTLILTEGDSAKSLAVAGMAVIGRDYYGCFPLRGKLLNVRDASTEQIAKNAEITA
Candida auris	CTLILTEGDSAKSLAVAGLSVVGRDYYGCFPLRGKLLNVRDASTEMIAKNAEITA
Candida haemulonii	CTLILTEGDSAKSLAVAGFSVVGRDYYGCFPLRGKLLNVRDASTEMIAKNAEITA
Meyerozyma guilliermondii	CTLILTEGDSAMPLAVAGLTVVGRDYYGCFPLRGKLLNVREASIEQVSKNAEINS
Candida parapsilosis	CTLILTEGLSALNLAVAGLTVIGRDYYGCFPLRGKLLNVREASADQIAKNAEINS
Candida orthopsilosis	CTLILTEGLSALNLAVAGLTVIGRDYYGCFPLRGKLLNVREASADQIAKNAEINS
Candida tropicalis	CTLILTEGLSALNLAVAGLSVVGRDYYGCFPLRGKLLNVREASADQISKNAEINA
Candida albicans	CTLILTEGLSAMNLAVAGLSVVGRDYYGCFPLRGKLLNVREASADQISKNAEINS
Candida dubliniensis	CTLILTEGLSAMNLAVAGLSVVGRDYYGCFPLRGKLLNVREASADQIAKNSEINS
Schizosaccharomyces pombe	CVLILTEGDSAKSLAVSGLSVVGRDYYGVFPLRGKLLNVREASHSQILNNKEIQA
Fusarium oxysporum	CTLILTEGDSAKGLAVSGRAILDPDRIGVFPLRGKLLNVRDASVDQIMKNAEIQN
Aspergillus nidulans	CTLILTEGESAKGLAMAGRAVVGADLFGVYPLRGKMLNVRDASFDQISKNEEIQS
Paracoccidioides brasiliensis	CTLILTEGDSAKGLAMAGRAIVGPDLFGVFPLRGKMLNVRDASVDQISKNAEIQN
Blastomyces dermatitidis	CTLILTEGDSAKGLAMAGRAIVGPDLFGVFPLRGKLLNVRDASVDQISKNAEIQN
Histoplasma capsulatum	CTLILTEGDSAKGLAMAGRAIVGPDLFGVFPLRGKLLNVRDASVDQISKNAEIQN
Coccidioides immitis	CTLILTEGDSAKGLAMAGRAVVGPDLFGVFPLRGKLLNVRDASVDQISKNQEIQN
Penicillium marneffei	CTLILTEGDSAKGLAMAGRAVVGPDLFGVFPLRGKLLNVRDASVDQISKNAEIQN
Aspergillus niger	CTLILTEGDSAKGLAMAGRAVVGPDLFGVFPLRGKLLNVRDASFDQISKNQEIQN
Aspergillus terreus	CTLILTEGDSAKGLAMAGRAVVGPDLFGVFPLRGKLLNVRDASFDQIAKNQEIQN
Aspergillus clavatus	CTLILTEGDSAKGLAMAGRAVVGPDLFGVFPLRGKLLNVRDASFEQISKNQEIQN
Aspergillus fumigatus	CTLILTEGDSAKGLAMAGRAVVGPDLFGVFPLRGKLLNVRDASFEQISKNQEIQN
Ustilago maydis	CTLILTEGDSAKSLAVAGIVEVGRDNYGVFPLRGKLLNVREASHDQIMKNAEIKA
Cryptococcus neoformans var. neoformans	CTLILTEGDSAKALAVSGLAVVGRDEYGVFPLRGKLLNVREAGHDQIVKNVEIQH

Homo sapiens	IIKIVGLQYKKNYEDEDSLKTLRYGKIMIMTDQDQDGSHIKGLLINFIHHNWPSLLR-HRFLEE
Mus musculus	IIKIVGLQYKKNYEDEDSLKTLRYGKIMIMTDQDQDGSHIKGLLINFIHHNWPSLLR-HRFLEE
Rattus norvegicus	IIKIVGLQYKKNYEDEDSLKTLRYGKIMIMTDQDQDGSHIKGLLINFIHHNWPSLLR-HRFLEE
Yarrowia lipolytica	IKEIIGLQHKKHY--TSC-KDLRYGHLMIMTDQDHDGSHIKGLIINFLETMFPGLLQIPGFLLE
Candida utilis	LKQIVGLQHKKRY--EDT-KSLRYGRVMLMTDQDTDGSHIKGLLINFIESQWPELLKIPGFLLE
Candida krusei	IKQIMGLQHKKHYGPDDI-KSLRYGRIMIMADQDHDGSHIKGLLINFLEASFPGLLEIPNFLIE
Kluyveromyces marxianus	IKKIMGLQHKKRY--EDA-TSLRYGHIMIMTDQDHDGSHIKGLIINFLESSFPGLLDIPGFLIE
Saccharomyces cerevisiae	IKKIMGLQHRKKY--EDT-KSLRYGHLMIMTDQDHDGSHIKGLIINFLESSFPGLLDIQGFLLE
Candida glabrata	IKKIMGLQHRKKY--EDT-KSLRYGHLMIMTDQDHDGSHIKGLIINFIETSFPGLLDIPGFLIE
Clavispora lusitaniae	LKQIIGLQHRKHYTLENI-KDLRYGHVMIMTDQDHDGSHIKGLLINFFESSFPGLLNIPGFLLE
Candida auris	LKQIIGLQHRKAYNRDNI-KDLRYGHVMIMTDQDHDGSHIKGLLINFFETSFPGLLNIDEFLLE
Candida haemulonii	LKQIIGLQHRKVYNRDNI-KDLRYGHVMIMTDQDHDGSHIKGLLINFFETSFPGLLNIDEFLLE
Meyerozyma guilliermondii	IKQIMGLQHKKRYTPENI-KSLRYGHIMIMTDQDQDGSHIKGLIINFLETSFPGLLEIPGFLLE
Candida parapsilosis	LKQIIGLQHKKTYNAENI-KSLRYGHIMIMTDQDQDGSHIKGLIINFLETSFPGLLDIPGFLLE
Candida orthopsilosis	LKQIIGLQHKKAYTAENI-KSLRYGHIMIMTDQDQDGSHIKGLIINFLETSFPGLLDIPGFLLE
Candida tropicalis	LKQIIGLQHKKHYTAENI-KQLRYGHIMIMTDQDQDGSHIKGLIINFLETSFPGLLEIPGFLLE

Candida albicans	LKQIIGLQHKKVYTAENI-KSLRYGHIMIMTDQDQDGSHIKGLIINFLETSFPGLLDIPGFLLE
Candida dubliniensis	LKQIIGLQHKKVYTAENI-KSLRYGHIMIMTDQDQDGSHIKGLIINFLETSFPGLLDIPGFLLE
Schizosaccharomyces pombe	IKKIMGFTHKKTY--TDV-KGLRYGHLMIMTDQDHDGSHIKGLIINYLESSYPSLLQIPGFLIQ
Fusarium oxysporum	IKQFLGLKHKQTY--TDT-KNLRYGHLMIMADQDHDGSHIKGLLINFLQVQFPSLLKIPDFFRE
Aspergillus nidulans	IKNFMGLQHKKEY--TDT-KGLRYGHLMIMTDQDHDGSHIKGLLINFLEAQFPSLLKIPEFLIE
Paracoccidioides brasiliensis	IKNFLGLQHKKEY--TDT-KGLRYGHLMIMTDQDHDGSHIKGLLINFLQVAFPSLLKIPQFLIE
Blastomyces dermatitidis	IKNFLGLQHKKEY--TDT-KGLRYGHLMIMTDQDHDGSHIKGLLINFFQVAFPSLLKIPQFLIE
Histoplasma capsulatum	IKNFLGLQHKKEY--TDT-KGLRYGHLMIMTDQDHDGSHIKGLLINFFQVAFPSLLKIPQFLIE
Coccidioides immitis	IKNFLGLQHKKEY--TDT-RGLRYGHLMIMTDQDHDGSHIKGLLINYLQVAFPSLLKIPGFLIE
Penicillium marneffei	IKSFIGLQHKKEY--TDT-RGLRYGHLMIMTDQDHDGSHIKGLLINFLQVQFPSLLKIPEFLIE
Aspergillus niger	IKNFLGLQHKKEY--TDT-RGLRYGHLMIMTDQDHDGSHIKGLLINFLQAQFPSLLKIPEFLIE
Aspergillus terreus	IKNFIGLQHKKEY--TDT-RGLRYGHLMIMTDQDHDGSHIKGLLINFLQAQFPSLLKIPEFLIE
Aspergillus clavatus	IKNFMGLQHKKEY--IDT-RGLRYGHLMIMTDQDHDGSHIKGLLINFLQAQFPSLLKIPEFLIE
Aspergillus fumigatus	IKNFLGLQHKKEY--TDT-RGLRYGHLMIMTDQDHDGSHIKGLLINFLQAQFPSLLKIPEFLIE
Ustilago maydis	IKEILGLQHGKQY--LDT-NSLRYGSIMIMTDQDHDGSHIKGLIINFLDHFYPSLLKIPGFLVE
Cryptococcus neoformans var. neoformans	IKQILGLKHNQDY--ATA-DSLRYGHLMIMTDQDHDGSHIKGLIINFLDHFYPSLLRIPNFLVE

Figure S1. Alignment of the amino acid sequences of the active site region of the DNA topoisomerase I and II enzymes of the families of organisms under study. (**a**) The conserved domain of Topo I is indicated in green and the conserved motif in fuchsia. (b) The toprim domain is highlighted in green, including the aspartic acid, lysine, and asparagine residues. The two glutamate regions (E, K, N, and DxD) are illustrated in fuchsia.

(**a**) (**b**)

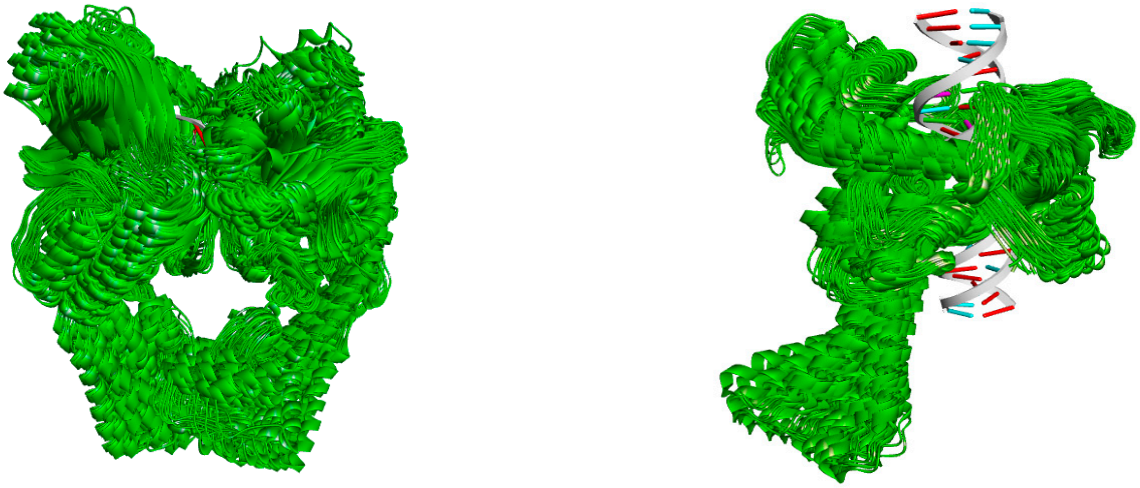

Figure S2. Overlapping of the 3D models from Topo I (a) and Topo II (b) of 29 fungal and 3 mammalian organisms (including *H. sapiens*). Models of the organisms in complex with DNA are shown in green.

(a)

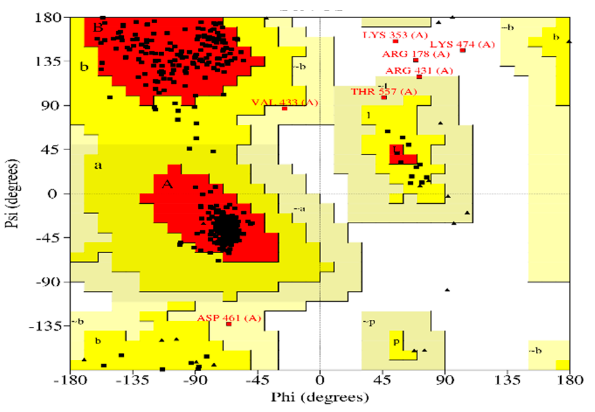

91.7%

(b)

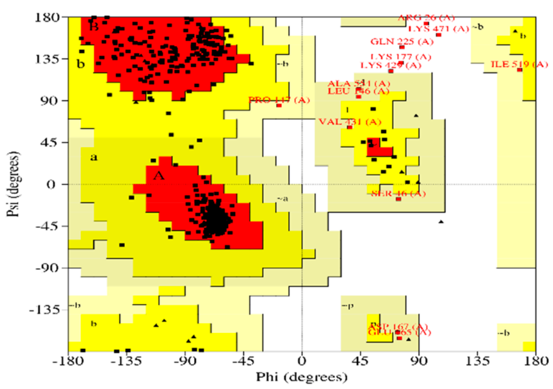

91%

(c)

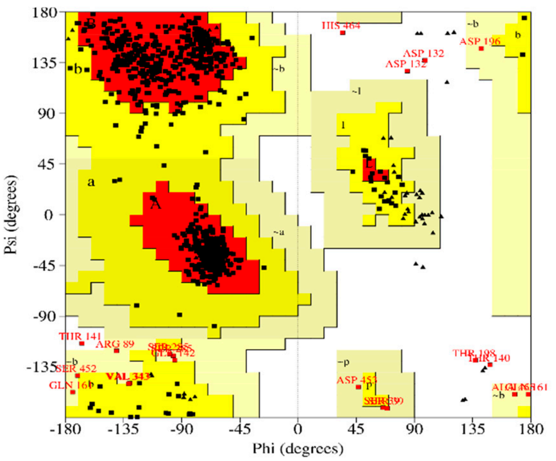

91.4%

(d)

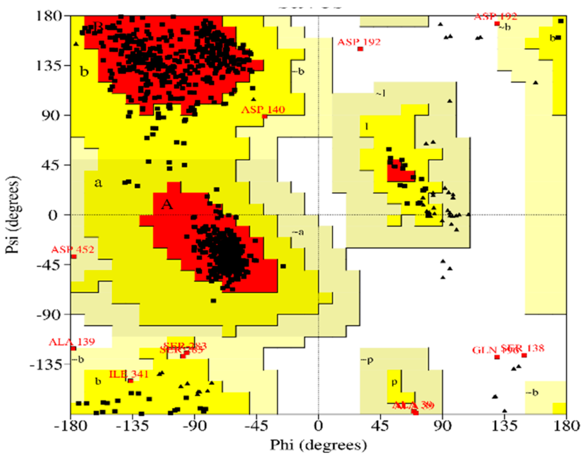

92.9%

Figure S3. Ramachandran plot of the models of Topo I (a-b) and Topo II (c-d) from representative organism (*C. auris* and *C. neoformans*, respectively). Allowed regions are shown in red, the less favorable regions in yellow, and the unfavorable regions in beige and white. The number indicates the percentage of amino acid residues that are located in favorable areas.

Table S1. Analysis of the identity and similarity of Topo I enzymes from mammals and fungi.

Organism	*H. sapiens* Identity/similarity %	*C. auris* Identity/similarity %	*C. neoformans* Identity/similarity %
Homo sapiens	100/100	39.5/53.4	39.5/53.7
Mus musculus	97/98.7	41.7/56.9	39.5/53.7
Rattus norvegicus	97/98.7	39.6/54	39.4/53.3
Saccharomyces cerevisiae	43/56.8	60.9/75.8	47.5/67.8
Schizosaccharomyces pombe	38.9/53.9	49.6/68.6	50/66.6
Yarrowia lipolytica	42.3/56.2	58.4/71.7	51.4/66.2
Candida albicans	40.1/56.3	65.6/78	49.4/65.7
Candida auris	39.7/53.6	100/100	45.3/63
Candida haemulonii	38.8/53.7	86.8/92.6	46.7/62.6
Clavispora lusitaniae	39.8/56.1	72.8/84.1	46/62.9
Candida dubliniensis	39.8/55.9	65.8/78.9	47.9/64.8
Candida tropicalis	40.8/56.1	66.1/79.7	48.9/66.5
Candida krusei	42.3/59.7	59.4/73.3	50.9/67.1
Kluyveromyces marxianus	39.9/54.7	59.8/75.8	43.4/59.9
Candida parapsilosis	39/54.5	66.1/78.6	46.5/62.6
Candida orthopsilosis	38.9/55	65.7/79.7	46.3/62.2
Candida glabrata	42.6/57.9	57.7/71.4	47.3/65.6
Candida utilis	41.1/54.4	62.4/77	48.3/64.7
Meyerozyma guilliermondii	41.2/56.1	64.5/80.5	48/63.5
Ustilago maydis	28.6/42.4	33/48.1	32.9/47.8
Coccidioides immitis	37.7/52.1	50.4/67	48.6/66.1
Aspergillus niger	38/52.9	50.6/67.2	48.8/64.5
Aspergillus clavatus	38.4/54.4	52/68.2	47.3/65
Aspergillus nidulans	37.8/54.5	52.9/67.8	49.5/66
Aspergillus terreus	36.6/53.7	51.6/67.4	48.6/65.7
Aspergillus fumigatus	38/54	51.5/67.5	48.6/65
Penicillium marneffei	40.2/54.2	52.8/67.8	50.7/68
Paracoccidioides brasiliensis	38.7/55.3	50.4/67	49.8/65.7
Fusarium oxysporum	38.7/53.5	48.7/65.7	47/61.1
Blastomyces dermatitidis	38.8/53	51.6/68.9	49.3/65.6
Histoplasma capsulatum	38/53.2	46.7/61	49.9/65.2
Cryptococcus neoformans var. neoformans	39.5/53.7	45.3/63	100/100

Table S2. Analysis of the identity and similarity of Topo II enzymes from mammals and fungi.

Organism	*H. sapiens* Identity/similarity %	*C. auris* Identity/similarity %	*C. neoformans* Identity/similarity %

Homo sapiens	100/100	43.8/60.5	53/68.7
Mus musculus	89.5/93.9	43/58.7	51.8/68.3
Rattus norvegicus	88.7/93.2	43/58.7	51.9/68.1
Saccharomyces cerevisiae	42.5/58.8	55.3/69.8	54.6/70.3
Schizosaccharomyces pombe	44.1/60.5	46.9/63.8	53.6769.4
Yarrowia lipolytica	41.6/58.1	51.5/66.1	52.8/66.7
Candida albicans	41.8/57.6	63.1/77.3	51.7/66.5
Candida auris	43.8/60.5	100/100	52.7/69
Candida haemulonii	42.9/60.8	84.1/90.9	52.4768.5
Clavispora lusitaniae	44.7/61.8	71/80.9	52.7/68.5
Candida dubliniensis	42.7/58.3	63.3/77.2	51.6/66.5
Candida tropicalis	47.7/63.9	64.9/78.4	51.9/68.4
Candida krusei	44/61.7	54.3/67.8	52.1/68.1
Kluyveromyces marxianus	43.4/58.4	55.3/70.2	53.8/70.1
Candida parapsilosis	45.9/62.6	61.8/75	52.7/67.9
Candida orthopsilosis	45.1/62.2	61.7/75.2	53.8/69.8
Candida glabrata	44.8/62.3	55.5/69.3	53.4/69.2
Candida utilis	45.3/61.8	53.5/67.6	52/68.1
Meyerozyma guilliermondii	43.5/59.5	64.5/78.5	53/68.7
Ustilago maydis	43.1/58.9	45.6/60.7	58.2/72.7
Coccidioides immitis	42.3/58.3	47.9/64.2	54.2/68.1
Aspergillus niger	42/58.6	47.9/64.6	54.8/69.9
Aspergillus clavatus	41.5/58.3	46.9/62.2	55/70
Aspergillus nidulans	40/56.8	44.8/62.1	52.70/68.5
Aspergillus terreus	41.3/57.4	48.6/64.2	54.6/69
Aspergillus fumigatus	42.47/58.7	47.4/62.6	55.9/71.2
Penicillium marneffei	43.2/58.8	48/64	53.2/67.2
Paracoccidioides brasiliensis	39.5/55.2	45.3/61.4	54.5/69.3
Fusarium oxysporum	40.6/56.7	45.6/61.2	52.5/66.6
Blastomyces dermatitidis	40.6/56.5	47.6/63.3	54.7/69.5
Histoplasma capsulatum	40.4/55.6	46.7/62.8	55.4/69.3
Cryptococcus neoformans var. neoformans	53/68.7	52.7/69	100/100

Table S3. RMSD value of Topo I and II 3D models of representative organisms.

**Topo I *C. auris***	0.515 Å
**Topo I *C. neoformans***	0.000 Å
**Topo II *C. auris***	1.08 Å
